# Osimertinib and anti-HER3 combination therapy engages immune dependent tumor toxicity via STING activation in trans

**DOI:** 10.1038/s41419-022-04701-3

**Published:** 2022-03-28

**Authors:** J. M. Vicencio, R. Evans, R. Green, Z. An, J. Deng, C. Treacy, R. Mustapha, J. Monypenny, C. Costoya, K. Lawler, K. Ng, K. De-Souza, O. Coban, V. Gomez, J. Clancy, S. H. Chen, A. Chalk, F. Wong, P. Gordon, C. Savage, C. Gomes, T. Pan, G. Alfano, L. Dolcetti, J. N. E. Chan, F. Flores-Borja, P. R. Barber, G. Weitsman, D. Sosnowska, E. Capone, S. Iacobelli, D. Hochhauser, J. A. Hartley, M. Parsons, J. N. Arnold, S. Ameer-Beg, S. A. Quezada, Y. Yarden, G. Sala, T. Ng

**Affiliations:** 1 Molecular Oncology Group, Cancer Institute, Paul O’Gorman Building, University College London, London, UK; 2 Richard Dimbleby Laboratory of Cancer Research, School of Cancer & Pharmaceutical Sciences, King’s College London, London, UK; 3 Cancer Immunology Unit, Cancer Institute, University College London, London, UK; 4 Wellcome Trust-MRC Institute of Metabolic Science, Addenbrooke’s Hospital, Cambridge, UK; 5 Centre for Immunobiology and Regenerative Medicine, Barts & The London School of Medicine and Dentistry, Queen Mary University of London, London, UK; 6 School of Cancer & Pharmaceutical Sciences, King’s College London, London, UK; 7 Department of Innovative Technologies in Medicine & Dentistry, University of Chieti-Pescara, Center for Advanced Studies and Technology (CAST), Chieti, Italy; 8 MediaPharma SRL, Chieti, Italy; 9 Randall Centre for Cell and Molecular Biophysics, King’s College London, London, UK; 10 Department of Biological Regulation, The Weizmann Institute of Science, Rehovot, Israel

**Keywords:** Non-small-cell lung cancer, Prognostic markers, Experimental models of disease, Preclinical research, Growth factor signalling

## Abstract

Over the past decade, immunotherapy delivered novel treatments for many cancer types. However, lung cancer still leads cancer mortality, and non-small-cell lung carcinoma patients with mutant EGFR cannot benefit from checkpoint inhibitors due to toxicity, relying only on palliative chemotherapy and the third-generation tyrosine kinase inhibitor (TKI) osimertinib. This new drug extends lifespan by 9-months *vs*. second-generation TKIs, but unfortunately, cancers relapse due to resistance mechanisms and the lack of antitumor immune responses. Here we explored the combination of osimertinib with anti-HER3 monoclonal antibodies and observed that the immune system contributed to eliminate tumor cells in mice and co-culture experiments using bone marrow-derived macrophages and human PBMCs. Osimertinib led to apoptosis of tumors but simultaneously, it triggered inositol-requiring-enzyme (IRE1α)-dependent HER3 upregulation, increased macrophage infiltration, and activated cGAS in cancer cells to produce cGAMP (detected by a lentivirally transduced STING activity biosensor), transactivating STING in macrophages. We sought to target osimertinib-induced HER3 upregulation with monoclonal antibodies, which engaged Fc receptor-dependent tumor elimination by macrophages, and STING agonists enhanced macrophage-mediated tumor elimination further. Thus, by engaging a tumor non-autonomous mechanism involving cGAS-STING and innate immunity, the combination of osimertinib and anti-HER3 antibodies could improve the limited therapeutic and stratification options for advanced stage lung cancer patients with mutant EGFR.

## Introduction

Lung cancer leads cancer-related mortality, but the discovery of oncogenic driver mutations improved treatments for metastatic non-small cell lung cancer (NSCLC). The epidermal growth factor receptor (EGFR, ErbB, HER) pathway affects most patients with targetable mutations. Kinase-activating EGFR-aberrations exist in 15-40% of NSCLC patients [1]. For this population, first or second-generation EGFR tyrosine kinase inhibitors (TKIs) like gefitinib, erlotinib and afatinib increase progression-free survival (PFS). A meta-analysis of trials involving gefitinib/erlotinib, showed superior PFS for TKIs *vs*. chemotherapy (11.0 vs. 5.6 months) [2]. However, TKI-resistance led to the development of osimertinib, a third-generation, irreversible EGFR-TKI selectively targeting both sensitising (exon-21 L858R) and gatekeeper (exon-20 T790M) mutations. The Phase III FLAURA trial evidenced longer PFS for osimertinib vs. early-generation TKIs in first-line setting (18.9 *vs*. 10.2 months) [3], a benefit sustained in updated overall survival (OS; median 38.6 vs. 31.8 months) [4]. An equivalent outcome reported the FLAURA-China trial, where osimertinib extended PFS by 8.0 months *vs*. comparator TKI [5]. This led to FDA and EMA approvals of osimertinib as first-line therapy for locally advanced or metastatic NSCLC with EGFR exon-19 deletions or exon-20 or -21 mutations (detected by an approved companion diagnostic test). Despite this, patients inevitably develop osimertinib-resistance, progressing without clear-cut stratification options other than chemotherapy. It is expected that new-generation TKIs targeting novel mutations will be required in future years, highlighting the urgent need to explore novel combination treatments based on rational patient stratification, to decrease relapse and increase lifespan.

Treatment resistance arises from tumor-intrinsic molecular mechanisms, allowing cancers to rewire their signalling pathways, feeding oncogenic drivers, and evading immune recognition. Cetuximab-mediated EGFR inhibition increases endoplasmic reticulum (ER) stress [6], which itself facilitates anticancer drug resistance [7], and mediates immune evasion through the IRE1α signalling axis [6, 8, 9]. Osimertinib resistance is highly heterogenous [10] and on-target mutations (e.g., exon-20 C797S) only account for a small percentage of these mechanisms [11], making it frequent to see other kinases or ErbB-family members upregulated post-osimertinib. HER3 overexpression per se correlates with metastatic progression and decreased relapse-free survival in NSCLC [12]. HER3 upregulation post osimertinib has been observed preclinically, and a triple combination of monoclonal antibodies (mAb) targeting HER1-2-3, combined with osimertinib worked synergistically against tumors, bypassing resistance mechanisms [13]. Recently in a Phase I dose-escalation/expansion study, patients with metastatic EGFR-mutant/NSCLC with prior TKI therapy (including osimertinib) showed clinical benefit following treatment with HER3-DXd (topoisomerase-I inhibitor-based HER3-ADC) [14]. Thus, HER3-targeting with mAbs appears as an important new candidate to treat TKI-resistant NSCLC, however current studies are restricted to targeting HER3 within tumor cells, without necessarily engaging an immune response.

Growing evidence supports the concept that micro-metastases and satellite tumor cells inevitably arise post-chemotherapy, and durable responses can only be achieved harnessing the immune system, with future treatment strategies needing alignment to that purpose. However, efforts to reproduce durable responses with immune checkpoint inhibitors (ICI) have failed in mutant-EGFR/NSCLC. Subgroup analyses of trials using ICIs in EGFR-mutant patients exhibited no PFS or OS benefit [15, 16], contrary to the EGFR^WT^ cohort. The combination osimertinib/durvalumab (anti-PD-L1) in Phase I trials produced impressive response rates but caused significant toxicity manifested as interstitial pneumonitis, resulting in study halting for safety reasons [17]. The option to target HER3 instead of immune checkpoint molecules is therefore attractive not only for the lower toxicity it could cause, but for the unexplored potential of activating an immune component through the interaction of the Fc-domain of antibodies with Fcγ-receptors (FcγR) present in innate immune cells.

NSCLC is characterised by extensive immune infiltrate co-opted by the growing tumor to promote an immunosuppressive microenvironment that allows tissue remodelling, thus facilitating metastasis. Most infiltrating immune cells are anti-inflammatory, pro-tumoral macrophages and their infiltration extent correlates with poor prognosis [18]. However, tumor-associated macrophages (TAM; or M2-like) can shift towards their classic pro-inflammatory phenotype (M1-like) via type-I IFN responses and activation of the cGAS/stimulator of interferon genes (STING) pathway [19], which is critical for initiating anticancer immune responses. STING agonists are being tested in clinical trials with the rationale of activating STING in TAMs to elicit immunostimulatory effects, alone or combined with established chemotherapy and immunotherapy [20]. Macrophages offer the additional advantage of contributing to antibody-based immunotherapy through FcγR-mediated effector activity [21–23], and STING improves the balance of activatory *vs*. inhibitory FcγR in TAMs [24].

There has been a paucity of research into the immunogenic effects of TKIs including osimertinib, particularly on innate immunity. An understanding of the influence of osimertinib on the immune landscape could illuminate further studies with combination therapies. Here we show that osimertinib activates STING in TAMs, which may have implications on downstream signals especially type-I IFN bridging innate and adaptive immunity. Using preclinical NSCLC models and state-of-the-art techniques, we show that osimertinib combined with anti-HER3 mAbs offers superior tumor control, and this is not just a synergistic effect of targeting EGFR/HER3 signalling simultaneously, but that HER3 mAbs also engage TAMs (via STING activation in trans), redirecting them to execute FcγR-mediated tumor cytotoxicity.

## Materials and methods

### Reagents

Osimertinib mesylate (AZD9291) was obtained through collaboration with AstraZeneca, Cambridge, UK. Monoclonal antibodies against human HER3 used for therapy were obtained via collaboration with Mediapharma S.R.L; MP-RM-1 is a murine-Fc monoclonal IgG2a against an extracellular epitope of human HER3 and EV20 is the humanised-Fc IgG1 version; both mAbs have been validated for in vitro and in vivo studies [25, 26]. Tunicamycin, Brefeldin-A, 3,3’-Dihexyloxacarbocyanine Iodide (DiOC_6_(3)), DAPI, PI were from Sigma Aldrich. CellEvent green cas3/7 and CellTracker orange CMTMR were from ThermoFisher Scientific. Cisplatin, DMXXA, 2’3’-cGAMP, Immobilion-F PVDF membranes were from Merk/Millipore. All CRISPR reagents were from Dharmacon (Horizon) as detailed in relevant sections. The antibodies used for confocal immunofluorescence in tumor sections were against HER3 (Cell signaling, #12708), F4/80-FITC and cGAS (#ab60343, #ab224144 from Abcam), STING-AF647 (RnD, #IC7169R); with secondary goat-anti-rabbit-AF546 (Invitrogen/ThermoFisher, #A11035). Antibodies used for westernblot were against HER3 (#12708), IRE1α (#3294), PERK (#5683), Bip (#3177), Chop (#2895), Xbp1s (#12792) from Cell Signaling Technology and against GAPDH (#CB1001) from Sigma-Aldrich/Merk/Millipore. The antibodies used for flow cytometry were against CD45 (#103132), Ly6c (#128041), Ly6g (#127628), NKp46 (#137621), CD107a/lamp1 (#121612), MHCII (#107648), F4/80 (#1231140), CD11b (#101226), as well as Zombie UV dye (#423107) from Biolegend; and against CD16/CD32 (from BD #563006), anti-CD32b (K9.361) and anti-FcγRIV (9E9.27) from the Ravetch laboratory through collaboration with S. Quezada. Unless otherwise stated, all other chemicals were from SigmaAldrich/Merk/Millipore.

### Cell lines and stable Cas9/CRISPR clone generation

H1975 and A549 cells were purchased from ATCC and cultured in RPMI and high-glucose DMEM respectively according to ATTC MTA; routine mycoplasma tests were conducted. Mediums were supplemented with 10% heat-inactivated FBS, penicillin/streptomycin and L-glutamine (SigmaAldrich). In brief, to generate stable H1975 NTC and IRE1α-ko cells, parental H1975 cells were electroporated with synthetic guide crRNAs, tracrRNA, recombinant Cas9 and mCherry plasmid; 72 h later, single mCherry^+^ cells were sorted into 96-well plates for clonal expansion; 3–6 weeks later, clonal phenotype was assessed for loss of IRE1α and functional validation. In detail, electroporation reactions were conducted using a Maxcyte STX instrument, program Opt-8. H1975 cells in P5 were trypsinised, washed twice in electroporation buffer (HyClone) and 3 × 10^6^ cells resuspended in 100 μl (per reaction) were added to disposable SOC100 electroporation cuvettes (Maxcyte) in a tissue culture hood. Each reaction contained 10 μg mCherry plasmid, 150 pmol of recombinant Cas9 (Dharmacon #CAS11200), 150 pmol of synthetic tracrRNA (Dharmacon #U-002005-20) and 150 pmol of either of the following guide synthetic crRNAs: Edit-R Non-Targeting Control #U-007501-01-20 or Edit-R Human ERN1 (2081) CM-004951-02-0002 from Dharmacon. After electroporation, all cells were taken out of SOC100 cuvettes and left to recover in incubator at 37 °C for 45 min in 6-well plates. Full medium was then added to each well and the cells were left in culture for gene editing and reporter expression. After 72 h, cells were trypsinised, washed, stained with DAPI for viability and single cells were plated into 96-well plates using a fluorescence-activated BD Aria Fusion cell sorter. To avoid artefacts due to transient mCherry expression in our clones, we sorted a low-intensity mCherry^+^ population (Supplementary Fig. S2A). Each clone was expanded for 3–6 weeks, and healthy growing clones were chosen for phenotyping IRE1α levels. Once clones #1 & #2 for crNTC and #4 & #5 for crIRE1α were identified (Supplementary Fig. 2B), we validated loss-of-function of IREα by treating cells with bona-fide ER-stressor tunicamycin 1 μg/ml for 3 h, and Xbp1s was quantified using near-infrared westernblot (Supplementary Figs. S2C and Fig. 2B).

### Human PBMC isolation

Human specimens were collected with written consent from volunteers in accordance with institutional review board guidelines and approval. Phlebotomy was conducted on healthy donors; 6–12 ml of peripheral blood were withdrawn into 6 ml tubes (BD Vacutainer® EDTA, purple cap) and spun down for 10 min at 1300 g at room temperature (RT) without centrifuge brake, this allowed plasma separation. The non-plasma fraction was diluted 1:1 with RPMI and carefully laid on top of Ficoll-Hypaque (GE Healthcare) solution (1 ml of Ficoll-Hypaque per 3 ml of blood/RPMI mixture) in separate 15 ml-tubes, and centrifugated at 800 g for 30 min at RT without brake in swinging-bucket rotor. The mononuclear cell layer was carefully extracted and washed 3 times with RPMI by 300 g centrifugation for 5 min at 4 °C. PBMCs were counted and used fresh for co-culture experiments or frozen by resuspension at 5 × 10^6^ cells/ml in 90% FBS/10% DMSO.

### Mice and in vivo studies

**Fig. 1:**
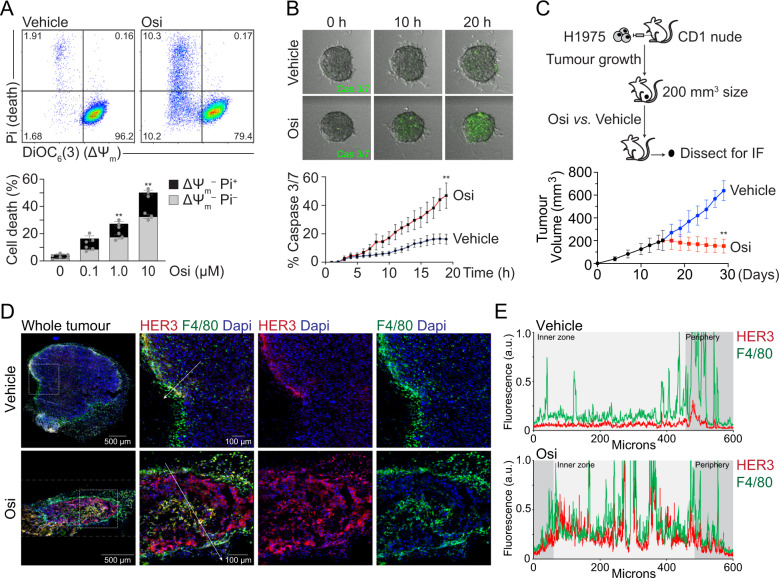
Osimertinib triggers apoptosis in 2D, 3D and in vivo models, leading to HER3 upregulation and macrophage tumour-infiltration. **A** H1975 cells were treated in 2D at the indicated osimertinib (Osi) concentrations for 24 h; apoptosis was measured using flow cytometry with DiOC_6_(3) for mitochondrial transmembrane potential (ΔΨ_m_) and PI for viability, as described in materials and methods. Representative dot plots show vehicle vs. 0.1 μM osimertinib-treated cells; the graph shows mean ± SEM of three experiments. **P < 0.01 *vs*. vehicle (0 μM) as indicated, using unpaired *t*-test with total values. **B** H1975 3D spheroids were treated with 200 nM osimertinib. The apoptotic activation of caspases 3/7 was monitored by fluorescence time-lapse microscopy. **P < 0.01 *vs*. vehicle as indicated using *t*-test; data shown as mean shows mean ± SEM of three replicate experiments. **C** In vivo xenograft diagram: 2.5 × 10^6^ H1975 cells were injected in 8 CD1-nude mice and tumor sizes were measured until 200 mm^3^-tumors were established. Vehicle *vs*. osimertinib oral *gavage* [5 mg/kg/day] groups were treated, and tumors were dissected after 10 days for immunofluorescence microscopy studies. The graph shows tumor growth curves, means ± SEM, **P < 0.01 *vs*. vehicle as indicated using *t*-test. **D** Confocal immunofluorescence images from xenograft tumor slices, showing HER3 distribution (red) and macrophages stained with F4/80 (green). Representative images are shown from 3 independent tumors; white scalebars as indicated. **E** Line-scan analysis of fluorescence intensities across the 600-μm-long white arrows from the merged channel images shown in D. For clarity, the inner *vs*. outer zones of tumors appear in the graphs as pale *vs*. darker grey respectively, showing increased HER3 levels and macrophage infiltration in the osimertinib-treated tumors. Graphs are representative from line-scans from 3 replicate tumors.

All animal studies were performed in accordance with institutional animal care and use committee guidelines and approval, in accordance with the local ethical review panel, the UK Home Office Animals Scientific Procedures Act 1986, and the UKCCCR guidelines. Female 6–8 week-old CD1-nude mice were purchased from Charles River UK and maintained in the New Hunt’s House BSU facilities, Guy’s campus, King’s College London. For in vivo studies, trial experiments were conducted on CD1-nude mice to assess response to osimertinib and to anti-HER3 mAbs MP-RM-1 and NG33 preliminarily [26, 27]. In these experiments 3 × 10^6^ H1975 cells were injected subcutaneously (*s.c*.) and tumor growth was monitored. For all other experiments (Figs. 1, 4), 2.5 × 10^6^ H1975 cells were injected *s.c*. and tumor growth was monitored until 200 mm^3^ size, then oral gavage osimertinib treatment started (2 mg/kg/day), and tumor volumes were determined using a digital calliper. For gavage we used 0.5% w/v hydroxyl propyl methyl cellulose in deionised water as vehicle. Osimertinib mesylate salt needs a 1.19 factor correction over osimertinib free form, hence a stock formulation at 29.75 mg/ml was prepared in vehicle and used for further dilutions as necessary. Stock was prepared by brief sonication and stirring at a speed that produced a vortex without creating excess frothing for a minimum of 4 h or overnight to achieve a smooth even suspension. Stock solution, stable for 10 days when continuously stirred at RT (not above 25 °C), was kept in amber vials protected from light. A lower concentration daily dose formulation, stable for up to 7 days in constant stirring, was prepared weekly. For the 2 mg/kg/day dose, formulations were made at 0.5 mg/ml. After animal dosing, formulation was returned to stirring. Treatment with anti-HER3 antibodies was via *i.p*. injections every 3 days using MP-RM-1 (10 mg/kg using PBS for vehicle control conditions).

### Murine bone marrow derived macrophages isolation and polarization

Bone marrow derived macrophages were obtained as previously described [28, 29]. A bone marrow cell suspension was obtained by flushing out femurs and tibias from CD1-nude mice with RPMI (supplemented with penicillin/streptomycin, L-glutamine and 10% FCS). Cells were washed 3× with PBS, resuspended in Red Blood Cell Lysing Buffer Hybri-Max (Sigma) for 5 min at RT, then washed 3× with PBS, counted and 2 × 10^6^ cells were seeded in P100 uncoated petri dishes. Macrophages were differentiated for 7 days by adding recombinant murine M-CSF (100 ng/ml) and polarized for further 24 h into M1-like (10 ng/ml M-CSF, 100 ng/ml IFNγ, 10 ng/ml LPS) or M2-like (10 ng/ml M-CSF, 20 ng/ml IL-4) for 24 h. All recombinant cytokines were from Peprotech and LPS from InvivoGen, UK. STING agonist 2’3’-cGAMP (1 μM, #531889, Sigma/Millipore) was added during the 24 h polarization period to M1-like differentiating macrophages for use in ELISA and macrophage-mediated cytotoxicity experiments shown in Fig. 7.

### Cytotoxicity and cell death assays for cell lines and co-culture experiments

H1975 or A549 cells were treated as indicated, then we used a flow cytometry protocol to measure early-apoptotic decay in mitochondrial transmembrane potential (ΔΨ_m_) and late-apoptotic plasma membrane permeabilization [30, 31]. 5 × 10^5^ H1975 or A549 cells were seeded in 12-well plates; after treatments, supernatants with non-adherent cells were collected and mixed with the adherent cells after trypsinization, spun down (300 × g, 5 min), resuspended in normal medium containing 20 nM DiOC_6_(3) and incubated for 10 min at 37 °C in the dark, followed by addition of PI or DAPI for cell viability and analysed immediately using a flow cytometer (BD LSR II Fortessa or BD Accuri). Analysed cells were first singlet-selected, then cross or spider quadrants were defined for each experiment. The early (ΔΨ_m_^–^) and late apoptotic (Viability dye^+^) populations were plotted together in gray and black bars as indicated with SEM values for each population. Statistics were assessed using the total apoptotic (early and late) values.

To measure exclusively the cell death of H1975 cells that were co-cultured with macrophages or PBMCs we adapted the above protocol, in which we pre-stained the macrophages or PBMCs with CellTracker orange CMTMR dye for 30 min prior to co-culture according to manufacturer’s instructions (Invitrogen/ThermoFisher, #C2927), and then excluded the macrophages from the gating analysis of cell death. Briefly, 1 × 10^6^ H1975 cells were plated in 6 well plates, treated with osimertinib for 20 h, then the anti-HER3 antibodies were added at concentrations indicated in the Figure legends for 1 h, to allow tumor cell opsonization. During this time, BMDMs or human PBMCs were stained with 1 μM CellTracker Orange CMTMR for 30 min, washed three times by centrifugation with PBS and counted. To assess FcγR implication we used a combination of blocking antibodies against CD16/CD32 (clone 2.4G2 from BD # 553141) and anti-FcγRIV (9E9.27) for 30 min in macrophages prior to co-culture. A ratio of 10:1 immune cells per H1975 cells was co-cultured for 2 h for cytotoxicity assays (or for 24 h for ELISA assays). Detached dead cells were collected and mixed with adherent cells after detaching using Enzyme-free Cell Dissociation Buffer (Gibco, #13151014). Cells were resuspended in full medium containing 20 nM DiOC for ΔΨ_m_, for 10 min at 37 °C in incubator. After this the vital dye DAPI was added, and cells were measured in a BD LSR II Fortessa (BD Biosciences). Analysis was conducted using FlowJo version 10.6.1. Analysed cells were first negatively gated on CMTMR (macrophages), singlet-selected, then cross or spider quadrants were defined for each experiment. The specific H1975 early (ΔΨ_m_^–^) and late (DAPI^+^) apoptotic populations were plotted as indicated with SEM values for each population. Statistics were assessed using the total apoptotic (early and late) values.

### Cryo-slicing, confocal immunofluorescence and ImageJ analysis

For cryo-sample preparation, fresh tumor tissues were collected and fixed in 4% PFA at 4 °C overnight, then samples were washed 3× in PBS and immersed in 30% sucrose at 4 °C until sample sunk to the bottom (or overnight). Specimens were then OCT-embedded in plastic moulds and snap frozen in ethanol and dry ice. Samples were transferred to -80 °C for long-term storage. For cryo-sections we used a Leica CM1950 cryostat set at 10-micron-thickness, slices were mounted on microscopy slides and stored at -80 °C until staining. For immunofluorescence, coverslips were taken from -80 °C and left to air dry at RT for 1 h, then samples were put in a jar incubated at 37 °C in water bath for 10 min, then washed 2× in PBS and fixed with 4% PFA, then washed 3× in PBS, permeabilized in 0.2% Triton-X100/PBS for 15 min and washed 3× in PBS, then blocked in 5% BSA/PBS for 1 h at RT. Primary antibodies at optimized dilutions in 5% BSA/PBS were incubated in the dark overnight at 4 °C in a humidified chamber, then washed 3× in PBS. Secondary antibodies at optimized dilutions in 5% BSA/PBS were incubated in the dark at RT for 1 h, then washed 3× in PBS and nuclear staining with DAPI was done for 20 min at RT. Extra fluorochrome-conjugated antibodies were incubated, post-secondary staining, prior to nuclear staining, in the dark overnight at 4 °C in a humidified chamber. Slices were then washed 3× in PBS and coverslips were mounted using Mowiol mounting medium; slices were allowed to air dry in the dark for 1 h, then imaged or were kept at 4 °C for storage. For imaging we used a Nikon A1 Point Scan 3.71 S inverted confocal in z-stack configuration and kept imaging settings equivalent between sample groups for consistency. Line scan fluorescence analysis (Fig. 1E and Supplementary Fig. S5B) was conducted using Fiji-ImageJ Dynamic Plot Profiler plugin keeping scan length consistency over channels using ROI manager tool. Immune cell infiltration (Fig. 5B) and STING^+^ cells (Fig. 5C) were quantified using Fiji-ImageJ Cell Counter plugin, over Dapi, F4/80 and STING channels. Surface HER3 imaging was done using confocal microscopy on H1975 Cas9/CRISPR cells in z-stack modality. Fixed (4% PFA, 10 min RT), non-permeabilized cells were stained with EV20 which targets an extracellular epitope of human HER3 (1 h, RT, 10 μg/ml), then stained with secondary anti-human-AF546 (Invitrogen/ThermoFisher, #A21089), and washed 3× in PBS; nuclei were stained with DAPI (15 min, RT) and coverslips were then mounted in slides using Mowiol mounting medium. HER3 quantification was conducted using Fiji-ImageJ defining cell edges as ROIs for both HER3 and cell area quantifications.

### Spheroid culture and timelapse imaging

Spheroids were cultured using the hanging drop method. Briefly, 75 × 10^3^ H1975 cells were suspended in 2.25 ml of 0.5%-FBS culture medium and mixed with 750 μl methylcellulose solution (0.2%). 30 μl drops were pipetted on the undersides of lids of 15 cm dishes using a multichannel pipette. The lids with hanging drops were put back onto PBS-containing dishes and cultured for 48 h to allow spheroid formation. Spheroids were then carefully transferred onto 96 well plates previously coated with 50 μL of collagen solution per well (25 mM Hepes, 1.6 mg/ml type-1 collagen, 17 mM NaOH, in Optimem). For transfer, 5 μl of spheroids were carefully taken from the hanging drops and placed on the top centre of the collagen layers and left to settle for 45 min in incubator; after this time Fluorobrite medium with apoptotic reporter dye (Cell event green caspase-3/7, according to manufacturer’s instructions; Thermofisher) in combination with vehicle (0.05% DMSO) or osimertinib (200 nM) was added for spheroid treatment. Plates were imaged live using a fast acquisition confocal Nikon Eclipse Ti inverted microscope equipped with a Yokogawa CSU-X1 disk head, an Andor Neo sCMOS camera. Fluorescence of the apoptotic reporter was quantified using Fiji ImageJ.

### ELISA

Supernatants from treated H1975 cells or co-cultured with BMDMs were centrifugated at 4 °C to remove debris and microvesicles by 500 × g (10 min) followed by 2 × 12200 × g (25 min) spins. Supernatants were then assessed for ELISA using VeriKine Human IFN multi-subtype kit or VeriKine Mouse IFN kit (RnD systems/PBL #41105 & #42120) according to manufaturer’s protocol. Plates were read at 450 nm using a Varioskan LUX multimode microplate reader (ThermoFisher).

### Near-infrared western blot

Cells were treated as indicated and lysed on ice using house-made buffer (100 mM tris, 300 mM NaCl, 0.5% v/v Nonidet-P40, Complete-Mini and Phospho-Stop cocktails (Roche) pH 7.4, 4 °C). Protein quantification was conducted with standard BCA staining and 50 µg of protein were loaded onto BisTris Nupage gels (10% or 4-12% from Invitrogen). Gels were transferred to PVDF Immobilion-F membranes (Millipore), blocked with 5% BSA for 45 min at RT, then incubated at 4 °C overnight with primary antibodies at optimized concentrations. Secondary near-infrared LICOR antibodies were used 1:20,000 in the dark for 1 h and membranes were washed and imaged using an Oddissey CLX scanner. Densitometry quantifications were made using ImageStudioLite from LICOR and normalized according to each experiment as indicated in Figure legends. Full-size blots are shown in supplementary data (Fig. S7).

### Flow cytometry measurements from tumors and cell lines

Dissected tumors were freshly cut into small pieces and resuspended in culture medium containing collagenase type 2 (1 mg/ml; Worthington Biochemical) and deoxyribonuclease I (0.1 mg/ml; Boehringer Mannheim), incubated at 37 °C with agitation for 1 h, and then filtered through a 40-mm mesh. Samples were centrifuged, washed twice with PBS, and stained for 30 min at room temperature. Antibodies used are detailed in section for reagents. For quantification of absolute number of cells, a defined number of fluorescent beads (Cell Sorting Set-up Beads for UV Lasers, ThermoFisher) was added to each sample before acquisition and used as a counting reference. Acquisition was performed with a BD LSR II Fortessa (BD Biosciences). Data analysis was conducted using FlowJo version 10.6.1 (Tree Star Inc.). For surface HER3 analysis, NTC and IRE1α-ko cells were treated with osimertinib 200 nM for 20 h and then resuspended with enzyme-free dissociation buffer (Gibco), washed 2× with cold PBS and incubated with EV20 (40 min on ice), washed 3× with cold PBS and stained with AF546-conjugated goat anti-human secondary (ThermoFisher, #A21089). Acquisition done with a BD LSR II Fortessa (BD Biosciences). Data analysis using FlowJo version 10.6.1 (Tree Star Inc.).

### Stable H1975-BioSTING inducible cells, Fluorescence lifetime imaging microscopy (FLIM) and Förster resonance energy transfer (FRET)

The 3^rd^ generation lentiviral BioSTING reporter construct [32] was donated by the Woodward laboratory (Department of Microbiology, UW Medicine at South Lake Union, Seattle, WA 98109, US). Upon receipt, dry plasmids were amplified using HiSpeed Plasmid Midi kit (QIAGEN #12643). The lentiviral transfer plasmid was then co-transfected into 293 T cells with the pRSV-Rev and pMDLg/pRRE packaging plasmids and the pCMV-VSV-G evelope plasmid using polyethylenimine reagent to generate lentiviral particles pseudo typed with VSV-G. H1975 cells were transduced with lentivirus and then subjected to puromycin selection over 10 passages to generate the BioSTING-H1975 stable cell line. BioSTING expression is controlled by a Tet-On system and was induced in 2.5 × 10^5^ BioSTING-H1975 cells plated on coverslips in 24-well plates via doxycycline treatment (0.5 μg/mL, 72 h). Cells were then treated with osimertinib (200 nM, 24 h) or 2’3’-cGAMP (1 μM, 1 h), washed 3× with PBS and fixed with 4% PFA (15 min, RT, washed 3×), permeabilised in 0.2% Triton-X100/PBS (15 min, washed 3×) and then treated with sodium borohydride/PBS (1 mg/ml, 15 min, RT; SigmaAldrich #71320) to eliminate PFA-induced autofluorescence. Coverslips were transferred onto microscopy glass slides using Mowiol mounting medium and allowed to air dry for 1 h in the dark. Samples were imaged using a multiphoton-FLIM TCSPC (Time-Correlated Single Photon Counting) imaging system [33]. Briefly, the FLIM system was built around a Nikon Eclipse Ti-E microscope fitted with a 40 × 1.30 NA Nikon Plan-Fluor oil objective and a 80 MHz Ti:Sapphire laser (Chameleon Vision II, Coherent) tuned to 875 nm (2-photon excitation wavelength for the donor mTFP1). Photons were collected using a 480 ± 30 nm emission filter (Semrock™) and an HPM 100-40 hybrid detector (Becker & Hickl). Laser power was adjusted to give average photon counting rates of the order 10^4^ to 10^5^ photons s^−1^ with peak rates approaching 10^6^ photons s^−1^. Acquisition times of the order of 300 s at low excitation power were used to achieve sufficient photon statistics for fitting while avoiding either pulse pile-up or significant photobleaching. All FLIM data were analysed using TRI2 [34], a time-resolved image analysis package, and were fitted with a mono-exponential Levenberg-Marquardt model. The data was further processed using a Python script to produce graphical representations of the fluorescence lifetime and FRET efficiencies using the following equation as previously described [35]:

$$\eta _{fret} = \left( {\frac{{R_0^6}}{{R_0^6 + r^6}}} \right) = 1 - \frac{{\tau _{fret}}}{{\tau _d}}$$

### Statistical analysis

All in vitro experiments were conducted with triplicate values from a min sample size of 3 independent repetitions; for co-culture experiments independent repetitions were considered as performed with BMDM coming from independent preparations. For in vivo studies, power calculations were obtained using G Power version 3.1.9; no changes in sample size were needed to be applied in this study based on pre-established inclusion/exclusion criteria according to local animal welfare regulations and licence in place. Randomization to allocated experimental animal groups was applied via blinded animal allocation done by independent staff to the researcher conducting treatments, with a 100% extent of blinding per treatment sessions. Quantification of statistical significance was calculated using GraphPad Prism 9.2.0 (283). Student’s *t* test was conducted for control vs. treatment studies and one-way ANOVA with Tukey’s test was conducted for multiple group tests with one categorical independent variable. For FLIM pixel frequency analysis we used Kolmogrov-Smirnov 2 sample test. Details for each statistical test indicated in Figure legends.

## Results

### Osimertinib triggers apoptosis in 2D, 3D and in vivo models, leading to HER3 upregulation and TAM infiltration

**Fig. 2:**
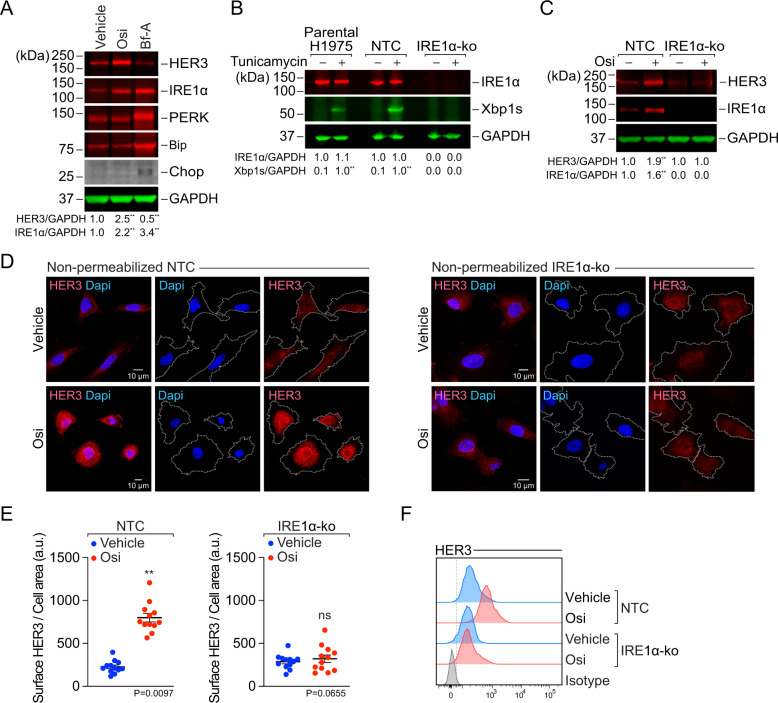
IRE1α is required for osimertinib to trigger HER3 upregulation in the cell surface. **A** Near-infrared quantitative westernblot analysis of H1975 cells treated with 200 nM osimertinib (Osi) or ER-stressor brefeldin-A (Bf-A, 0.5 μg/ml) for 24 h. Densitometry levels of HER3 or IRE1α relative to GAPDH were quantified from 3 independent replicates, and are shown under the representative blot lanes as normalized mean values (a.u.), where **P < 0.01 *vs*. vehicle is shown, as calculated using unpaired *t*-test. Changes in PERK, Bip and Chop levels after Osi were non-significant. Full-size blots are in supplementary data. **B** Parental H1975 cells, or Cas9/CRISPR stable non-targeting control (NTC) and IRE1α-ko H1975 cells were treated with ER-stressor tunicamycin (1 μg/ml) for 3 h to assess functionality of IRE1α. Densitometry levels of IRE1α or spliced-Xbp1 (Xbp1s) relative to GAPDH were quantified from 3 independent replicates (a.u.), and are shown under the representative blot lanes as mean values, internally-normalized per cell type; **P < 0.01 *vs*. vehicle as calculated using unpaired *t*-test. Full-size blots are shown in supplementary data. **C** Cas9/CRISPR stable NTC or IRE1α-ko H1975 cells were treated with 200 nM osimertinib for 24 h. Densitometry levels of HER3 or IRE1α relative to GAPDH were quantified from 3 independent replicates and are shown as mean values (a.u.) under the representative blot lanes, normalized per cell type; **P < 0.01 *vs*. vehicle is shown where pertinent, as calculated using unpaired *t*-test. Full-size blots shown in supplementary data. **D** Cas9/CRISPR stable NTC or IRE1α-ko H1975 cells were treated with 200 nM osimertinib (Osi) or vehicle for 24 h and processed for cell surface staining and confocal imaging as described in materials and methods, using an antibody targeting an extracellular epitope of HER3. Images showing surface HER3 (red) and nuclei (dapi, blue) are representative of 4 independent replicates; cell borders were obtained using Fiji-ImageJ and used for cell area quantifications shown in panel **E**. **E** Surface HER3 levels relative to cell area were quantified from 4 independent experiments including the images shown in panel **D** (total 3 cells per repetition, 12 data points). Data are shown as means ± SEM. **P < 0.01 *vs*. vehicle as indicated, using *t*-test. **F** Flow cytometry quantification of surface HER3 levels on live H1975 cells (NTC and IRE1α-ko clones) treated with vehicle (blue) or osimertinib (Osi, 200 nM, red) for 24 h, as described in materials and methods, stained using an antibody against an extracellular epitope of HER3. Histograms are representative of 3 independent replicates.

Osimertinib was administered at different doses to EGFR^L858R–T790M^ double-mutant H1975 cells and to EGFR^WT^ A549 NSCLC cells in 2D experiments. Apoptosis was measured by flow cytometry using markers for viability and mitochondrial-transmembrane potential (ΔΨ_m_) decay (Figs. 1A and S1). Unlike cisplatin (Fig. S1A, B), osimertinib caused higher apoptosis in H1975 *vs*. A549 cells as expected (Fig. S1C, D), due to the intrinsic cisplatin-resistance of H1975 cells and to the higher binding affinity of osimertinib towards EGFR^L858R–T790M^ *vs*. EGFR^WT^ [36, 37]. 3D experiments additionally revealed early-activation of caspases 3/7 in H1975 spheroids (Fig. 1B) confirming apoptosis. We further conducted in vivo H1975 xenograft experiments in CD1-nude mice, where osimertinib decreased tumor size (Fig. 1C, D). Ex vivo tumor immunofluorescence revealed HER3-upregulation post-osimertinib (Fig. 1D, E). Interestingly, whereas macrophages (F4/80-labelled) were mostly in the periphery of vehicle-treated tumors, osimertinib increased TAM infiltration into inner zones of tumors (Fig. 1D), towards HER3-high areas (Fig. 1D, E). Using westernblot, we validated HER3-upregulation post-osimertinib in vitro in H1975 cells (Figs. S1E and Fig. 2A).

### HER3 upregulation by osimertinib requires IRE1α

To mechanistically understand HER3 upregulation post-osimertinib, we explored cellular stress pathways triggered by osimertinib in cells. We found that the inositol-requiring enzyme (IRE1α)-branch of the unfolded protein response (UPR) was upregulated, whereas the protein kinase RNA-like ER kinase (PERK) or activating transcription factor 6 (ATF6)-dependent binding immunoglobulin protein Bip (Grp78, HSPA5) were not changed (Fig. 2A). Importantly, this was not accompanied by upregulation of the UPR death effector C/EBP-homologous (Chop), as it was after treatment with ER-stressor brefeldin-A, indicating that IRE1α activation was not a terminal ER stress response, and rather a non-lethal adaptive response to osimertinib. Based on this, we used Cas9/CRISPR to generate stable IRE1α-ko H1975 cells (Fig. S2A, B). We functionally validated IRE1α-ko cells assessing their inability to splice Xbp1 in response to bona fide ER-stressor tunicamycin (Figs. 2B, S2C). In IRE1α-ko cells osimertinib treatment was unable to upregulate HER3 compared to non-targeting control (NTC) cells (Fig. 2C). To evaluate whether HER3-upregulation correlated with exposure of this receptor on the cell surface, we performed immunofluorescence imaging on non-permeabilised cells using an antibody against an extracellular epitope of HER3 [25]. Osimertinib increased surface HER3 in NTC cells, but not in IRE1α-ko H1975 cells (Fig. 2D, E). We validated this using live cell flow cytometry, again showing increased surface HER3 post-osimertinib in NTC, not in IRE1α-ko cells (Fig. 2F). These results suggest that upregulation of surface HER3 could represent an adaptation to drug-induced stress, raising the possibility of targeting HER3 in the osimertinib-challenged tumor.

### Combination of osimertinib with anti-HER3 antibodies engages macrophage-mediated cytotoxicity

**Fig. 3:**
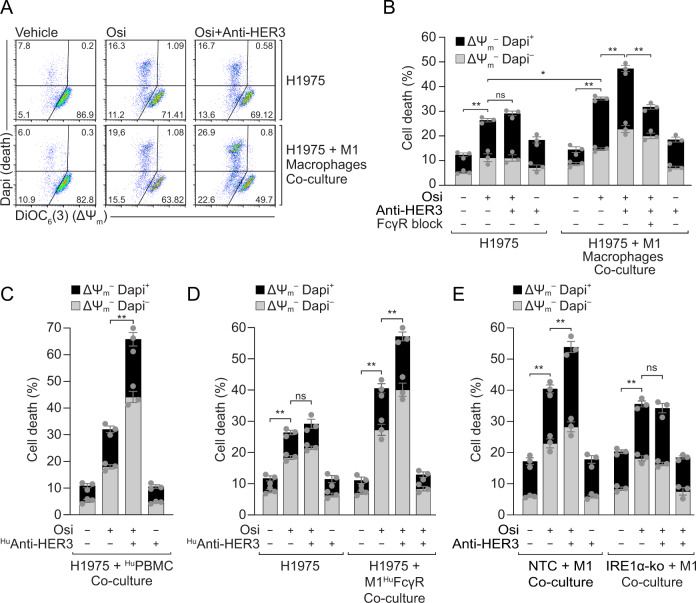
Combination therapy with osimertinib and anti-HER3 antibodies triggers macrophage-mediated FcγR-dependent cell toxicity. **A** H1975 cells pre-treated with osimertinib (Osi, 200 nM, 24 h) were treated with anti-HER3 (MP-RM-1, 10 μg/ml, 1 h) and then co-cultured with macrophages (2 h, ratio 10:1) pre-labelled with cell tracker. Apoptosis of H1975 cells was assessed by flow cytometry excluding bone marrow-derived macrophages (BMDM) as described in materials and methods. Dot plots are representative of 3 independent replicates. **B** Quantification summary from the representative data shown in panel **A**. H1975 cells alone or co-cultured with macrophages were assessed for apoptosis using flow cytometry with DiOC_6_(3) for mitochondrial transmembrane potential (ΔΨ_m_) and Dapi for cell viability, as described in materials and methods. FcγR block was added in co-culture conditions as indicated to assess FcγR function. Results shown as mean ± SEM from three replicates; *P < 0.05, **P < 0.01 as indicated, using one-way ANOVA with Tukey’s test. **C** H1975 cells pre-treated with osimertinib (200 nM, 24 h) were treated with human anti-HER3 (^Hu^Anti-HER3, EV20; 10 μg/ml, 1 h) and then co-cultured with healthy-donor derived human PBMCs (2 h, ratio 10:1). Apoptosis of H1975 cells was assessed by flow cytometry excluding immune cells as described in materials and methods. Data are shown as mean ± SEM. **P < 0.01 as indicated, using unpaired *t*-test with total values. **D** H1975 cells pre-treated with osimertinib (200 nM, 24 h) were treated with human anti-HER3 (^Hu^Anti-HER3, EV20; 10 μg/ml, 1 h) and then co-cultured with BMDM from chimeric humanised FcγR (^Hu^FcγR) mice (2 h, ratio 10:1) pre-labelled with cell tracker. Apoptosis of H1975 cells was assessed by flow cytometry excluding macrophages as described in materials and methods. Gray and black bars as in Panel **B**. Data are shown as mean ± SEM. **P < 0.01 as indicated, using unpaired *t*-test with total values. **E** Cas9/CRISPR stable NTC or IRE1α-ko H1975 cells pre-treated with osimertinib (200 nM, 24 h) were treated with murine anti-HER3 (MP-RM-1, 10 μg/ml, 1 h) and then co-cultured with M1-like BMDM from CD1-nude mice (M1; 2 h, ratio 10:1) pre-labelled with cell tracker. Apoptosis of H1975 cells was assessed by flow cytometry excluding macrophages as described in materials and methods. Gray and black bars as in Panel **B**. Data are shown as mean ± SEM. **P < 0.01 as indicated, using unpaired *t*-test with total values.

Based on the previous data, we explored whether combining osimertinib with anti-HER3 mAbs would lead to increased cell death. First, we used H1975 cells and MP-RM-1 [26], a murine IgG2a against human HER3, but no significant effect was observed following MP-RM-1 addition to osimertinib-treated cells in 2D experiments in vitro (Fig. 3A, B). We then co-cultured tumor cells with macrophages to assess whether they would contribute to H1975-cell killing. To this aim we labelled macrophages prior to co-culture (as detailed in methods) and excluded them from the flow cytometry apoptotic analysis, thus allowing us to address the cytotoxic effect of macrophages over opsonized H1975 cells. Consistent with the xenograft data from Fig. 1D, when H1975 cells were co-cultured with bone marrow-derived macrophages (BMDM) from CD1-nude mice, a significant increase in apoptosis of H1975 cells was osberved, especially after the combination therapy (Fig. 3A, B). The addition of an FcγR block abrogated the effects of adding the anti-HER3, suggesting that FcγRs mediated this response (Fig. 3B). Of note, these effects were only observed with BMDMs pre-skewed to an M1-like phenotype and were not observed with M0 or M2-like macrophages (Fig. S3A, B). To corroborate these results in a human-human model, we co-cultured H1975 cells with donor-derived human PBMCs and combined osimertinib with the humanised IgG1 anti-HER3, EV20 [25], where the combination treatment significantly increased tumor cell elimination (Fig. 3C). As these results directly implicated an interaction between the Fc-domain of anti-HER3 antibodies and the FcγRs from immune cells as a critical component of this response, we used BMDMs obtained from an immunocompetent chimeric mouse model in which murine FcγRs have been knocked-out and replaced by human functional FcγRs [38]. In this system we also observed synergy between osimertinib and the anti-HER3 EV20 (Figs. 3D, S4A, B). To further validate the role of HER3-upregulation in the response to the combination therapy, we co-cultured NTC and IRE1α-ko cells with CD1-nude-derived BMDMs. In IRE1α-ko cells, which fail to upregulate HER3 post-osimertinib (Fig. 2C–F), the effects of adding an anti-HER3 were abrogated (Figs. 3E, S4C); thus HER3-upregulation, as part of the resistance response to osimertinib, is crucial for the success of the mAb therapy. These results together, suggested that combining osimertinib with anti-HER3 antibodies elicits macrophage-dependent FcγR-mediated cytotoxicity, potentially increasing the engagement of innate immunity over osimertinib alone.

### Anti-HER3 antibody treatment boosts macrophage infiltration over osimertinib in vivo

**Fig. 4:**
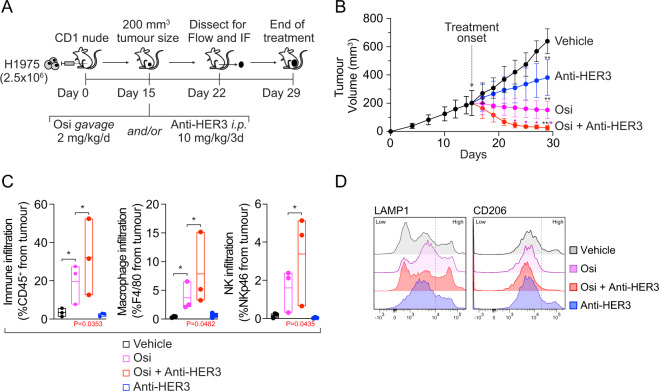
Combination therapy with osimertinib and anti-HER3 antibodies shows increased efficacy and immune engagement in vivo. **A** Diagram illustrating in vivo xenograft experimental design; 2.5 × 10^6^ H1975 cells were injected *s.c*. into CD1-nude mice. Once tumor volumes reached 200 mm^3^ (day 15), mice were divided into 4 groups and treatments begun as indicated, with daily osimertinib (Osi) or vehicle (hydroxyl propyl methyl cellulose) oral *gavage* and/or *i.p*. injections every 3 days (PBS or Anti-HER3, MP-RM-1). After 7 days (day 22), 6 mice per group were collected for tumor analysis, *n* = 3 for flow cytometry and *n* = 3 for immunofluorescence (IF) microscopy studies; the *n* = 6 remaining mice per group were kept for tumor size monitoring until day 29. **B**. Tumor volumes are shown for each group as indicated, mean ± SD. Statistical significance with one-way ANOVA with Tukey’s multiple group comparison test revealed *P < 0.05 and **P < 0.01, indicated above each tumor group, black asterisks are relative to vehicle group and magenta asterisks are relative to Osi group at the timepoints indicated. **C** Quantification of immune infiltration (CD45^+^, F4/80^+^ and NKp46^+^) using flow cytometry from tumors dissected from treatment groups defined in panel **A**. Data shown as floating boxes (mean ± interval) including datapoints. *P < 0.05 as indicated, using one-way ANOVA with Tukey’s multiple group comparison test with individual *P* values shown in red comparing Osi *vs*. Osi + Anti-HER3 groups. **D** Overlay histogram plots comparing marker expression (LAMP1 and CD206 low and high populations) in tumor infiltrating macrophages. Graphs are representative from individual treatment groups as detailed in panel **A**.

We further studied the osimertinib/anti-HER3 combination therapy in vivo using H1975 xenografts in CD1-nude mice. After tumor establishment (200 mm^3^, 15 days), mice were treated with osimertinib or vehicle *gavage*, in combination with *i.p*. injections of vehicle PBS or anti-HER3 MP-RM1 (Fig. 4A). We harvested tumors for microscopy and flow cytometry studies after 1-week treatment, before tumor sizes were too small for analysis (according to preliminary experiments); the remaining cohort received treatment until experimental end. Both the anti-HER3 and osimertinib groups themselves exhibited significant tumor size reduction compared with vehicle, but the benefit of the combination therapy was higher and was reached earlier, showing significant tumor volume reduction *vs*. osimertinib monotherapy at 8-days post-treatment (Fig. 4B). Flow cytometry tumor analysis revealed increased CD45^+^ immune infiltration, and increased macrophage (CD45^+^ F4/80^+^) and NK (CD45^+^ NKp46) infiltration (Fig. 4C), but not a significant increase in infiltrating CD45^+^ CD11b^+^ cells (Fig. S5A), consistent with a degranulation phenotype rather than an antigen presenting phenotype. Accordingly, the combination therapy increased LAMP1^Hi^ macrophage infiltration, consistent with degranulation, but decreased CD206^Hi^ macrophages, suggesting a decreased M2-like phenotype (Fig. 4D). These results further validated in vivo that the combination of osimertinib and anti-HER3 antibodies increases macrophage-dependent tumor control and could imply TAM-redirection towards an inflammatory phenotype.

### Combination treatment activates tumoral cGAS transactivating STING in macrophages

**Fig. 5:**
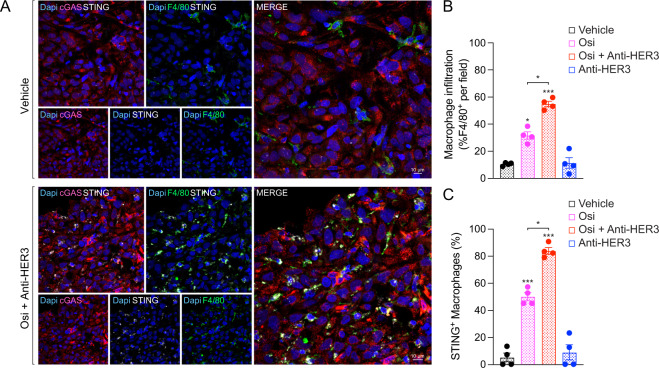
Osimertinib and anti-HER3 therapy combination increases STING in tumor infiltrating macrophages. **A** Confocal immunofluorescence images from xenograft tumor slices, showing cGAS distribution (red), infiltrating macrophages (F4/80, green), STING (white) and nuclei (Dapi, blue). Images are representative from treatment groups indicated (defined in Fig. 4A; all groups shown in Supplementary Fig. S6). White scalebars are indicated in merged channel images. **B** Infiltrating macrophages (F4/80^+^) were quantified *vs*. total cell number (Dapi) using Fiji-ImageJ. Data shown as mean ± SEM from image fields corresponding to the treatment groups indicated (defined in Fig. 4A). Statistical analysis using one-way ANOVA with Tukey’s multiple group comparison test revealed *P < 0.05 and ***P < 0.001 relative to vehicle or as indicated. **C** STING-positive macrophages (STING^+^ and F4/80^+^) were quantified *vs*. total macrophage number (F4/80^+^) using Fiji-ImageJ. Data shown as mean ± SEM from 4 independent image fields coming from the treatment groups indicated (defined in Fig. 4A). Statistical analysis using one-way ANOVA with Tukey’s multiple group comparison test revealed ***P < 0.001 relative to vehicle or *P < 0.05 and as indicated.

The cGAS/stimulator of interferon genes (STING) pathway is known to shift the pro-tumoral M2-like macrophage phenotype towards an inflammatory M1-like phenotype through type-I IFN responses [19]. STING also favours antibody therapy through enhancing FcγR-dependent macrophage-mediated tumor toxicity [24]. We studied this pathway to better understand the functional relationship between osimertinib-treated tumors and infiltrating macrophages. Using confocal immunofluorescence from in vivo xenografts (Fig. 4A), we observed cGAS as diffuse puncta spread throughout tumor cells under vehicle-treated conditions, which aggregated to form cGAS foci in osimertinib-treated tumors, especially in the inner zone of tumors (Fig. S5B), suggesting cGAS activation as previously reported [39]. Interestingly in cryosections from osimertinib-treated tumors, STING was restricted to small-nucleated infiltrating immune cells, while it was excluded from the large-nucleated H1975 cells (Figs. S5B, S6A). To address this and ascertain whether the cGAS and STING signals were coming from H1975 cells and macrophages respectively, we co-stained with F4/80 and observed that the increase in perinuclear STING was exclusively restricted to macrophages, particularly after the combination osimertinib/anti-HER3 (Fig. 5A–C and S6A–C), suggesting that macrophage STING could be activated because of tumor-cell cGAS activation post-osimertinib. Accordingly, STING^+^ macrophages were not seen after treatment with anti-HER3 only (Fig. S6A–C).

### Tumor cell-derived cGAMP leads to type-I interferon production by macrophages

**Fig. 6:**
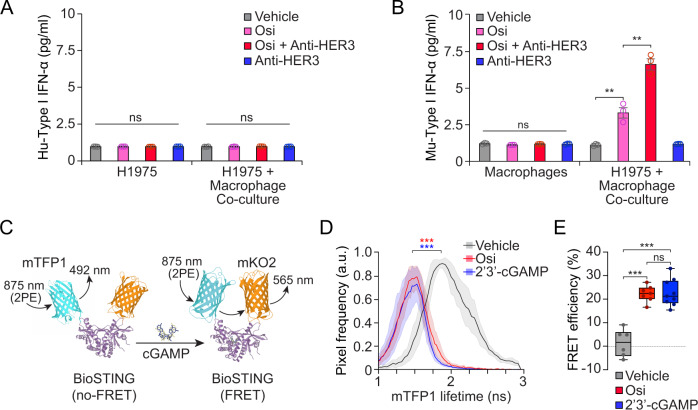
Osimertinib increases cGAMP production in tumor cells leading to type-I IFN production in macrophages. **A** Quantification of H1975-derived human type-I IFN. H1975 cells pre-treated with osimertinib (200 nM, 24 h) were treated with anti-HER3 (MP-RM-1, 10 μg/ml, 1 h) and then either co-cultured with bone marrow derived macrophages (BMDMs, 24 h, ratio 10:1) or maintained for 24 h; supernatants were assessed for ELISA as described in materials and methods. Data are shown as mean ± SEM from 3 independent experiments. Non-significant (ns) differences calculated using unpaired *t*-test. **B** Quantification of macrophage-derived murine type-I IFN. Supernatants from macrophages treated as indicated (24 h) or co-cultured with H1975 cells as described for Panel **A**, were assessed for ELISA as described in materials and methods. Data shown as mean ± SEM from 3 independent experiments. **P < 0.01 as indicated, using unpaired *t*-test. **C** Diagram illustrating the recombinant biosensor BioSTING, composed of cyclic di-nucleotide (CDN) binding domain (CBD, purple) of STING linked to teal fluorescent protein (mTFP1 at L152) and to orange fluorescent protein (mKO2 at E335), which upon 2-photon excitation (2PE) undergoes Förster resonance energy transfer (FRET) when bound to the CDN cGAMP. **D** H1975 cells stably expressing BioSTING were treated with osimertinib (200 nM, 24 h) or with CDN 2’3’-cGAMP (1 μM, 1 h) and then assessed for fluorescence lifetime imaging microscopy (FLIM) as described in materials and methods. Fluorescence lifetime of the donor (mTFP1) decreases as a result of FRET; the traces show frequency of pixel distribution *vs*. lifetime (ns), average values ± 95% confidence interval shown as lines and spectrum respectively. Data generated from 6 single cell acquisitions per condition (min) was used to generate a normalised summed plot. ***P < 0.001 as indicated by colour *vs*. vehicle, using Kolmogrov-Smirnov 2 sample test. **E** Quantification of FRET efficiency from the experiments described in panel D, indicating cGAMP production in H1975 cells after osimertinib treatment. Data shown as box plots of FRET efficiencies (median ± IQR & interval whiskers). ***P < 0.001 as indicated, using unpaired *t*-test.

Growing evidence indicates that cells undergoing DNA damage or chemotherapeutic stress activate cGAS to produce cyclic-GMP-AMP (cGAMP) leading to noncanonical STING transactivation in surrounding innate immune cells [40, 41]. Therefore, to assess the interplay between tumoral cGAS and macrophage STING further in our setting, we conducted in vitro experiments with H1975 cells alone, macrophages alone, or their co-cultures, analysing the supernatant of different conditions using species-specific ELISA to detect IFN from either human origin (H1975) or murine origin (macrophages), this allowed us to pinpoint the source of IFN production. We observed that H1975 cells did not produce human type-I IFN after osimertinib or the osimertinib/anti-HER3 combination; and co-culturing H1975 cells with macrophages did not change this (Fig. 6A). Similarly in treated macrophages, osimertinib did not cause murine type-I IFN production, however, when macrophages were co-cultured with osimertinib-treated tumor cells, we observed a significant increase in type-I IFN production by macrophages (Fig. 6B), suggesting that macrophages require tumor cells as a source of cGAMP to activate STING. To validate that osimertinib leads to cGAS activation and cGAMP production within tumor cells, we generated H1975 cells stably expressing a biosensor called BioSTING (Fig. 6C). This biosensor is composed of the cyclic-dinucleotide binding-domain (CBD) of STING coupled to the fluorescent donor mTFP1 and the acceptor mKO2; and undergoes FRET when the CBD binds its ligand 2’3’-cGAMP [32]. Using fluorescence lifetime imaging microscopy (FLIM), we detected a significant decrease in the lifetime of the donor mTFP1 post-osimertinib (Fig. 6D) which is expected after active FRET; accordingly, osimertinib triggered higher FRET efficiency (Fig. 6E). These combined results confirmed that osimertinib activates cGAS in tumor cells to produce cGAMP, promoting surrounding macrophages to increase STING-mediated type-I IFN responses.

### STING agonist 2’3’-cGAMP potentiates macrophage-mediated tumor cell toxicity

**Fig. 7:**
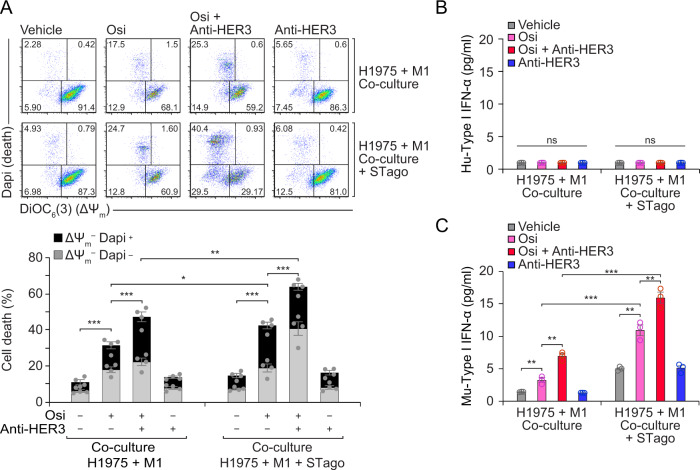
Addition of a STING agonist leads to increased type-I IFN production in macrophages and enhances macrophage-mediated tumor toxicity. **A** H1975 cells pre-treated with osimertinib (Osi, 200 nM, 24 h) and anti-HER3 (MP-RM-1, 10 μg/ml, 1 h) were co-cultured with M1-like bone marrow derived macrophages (BMDM) or BMDMs pre-treated with STING agonist (STago) 2’3’-cGAMP as indicated in materials and methods. Apoptosis of H1975 cells was assessed by flow cytometry excluding BMDMs; cells labelled with DiOC_6_(3) for mitochondrial transmembrane potential (ΔΨ_m_) and Dapi for cell viability, as described in materials and methods. Dot plots are representative of 3 independent replicates. A quantification summary is shown in the lower graph. Results shown as mean ± SEM from three replicates; ***P < 0.001, **P < 0.01 as indicated, using one-way ANOVA with Tukey’s test. **B** Quantification of H1975-derived human type-I IFN. H1975 cells pre-treated with osimertinib (Osi, 200 nM, 24 h) and anti-HER3 (MP-RM-1, 10 μg/ml, 1 h) were co-cultured for 24 h with M1-like bone marrow derived macrophages (BMDM) or BMDMs pre-treated with STING agonist (STago) 2’3’-cGAMP, then supernatants were assessed for ELISA as described in materials and methods. Data are shown as mean ± SEM. Non-significant (ns) differences quantified using unpaired *t*-test. **C** Quantification of macrophage-derived murine type-I IFN. H1975 cells were pre-treated with osimertinib and anti-HER3, then co-cultured with BMDMs as detailed for Panel B. Supernatants were assessed for ELISA as described in materials and methods. Data shown as mean ± SEM. ***P < 0.001, **P < 0.01 as indicated, using unpaired *t*-test.

We further studied the impact of externally adding the STING agonist 2’3’-cGAMP to macrophages, to assess their subsequent tumor cytotoxic capacity. The combination therapy osimertinib/anti-HER3 showed a maximum response in vivo (Fig. 4B), therefore we took an in vitro approach, where differences could be detected, using our co-culture model and flow cytometric apoptotic assessment of H1975 cells excluding macrophages. To this aim we pre-treated macrophages with 2’3’-cGAMP prior to co-culture; in line with our previous findings, macrophage-mediated cytotoxicity against H1975 increased significantly by pre-treating macrophages with 2’3-cGAMP (Fig. 7A). Using species-specific ELISA, we measured type-I IFN after co-culture, and observed that pre-treating macrophages with 2’3’-cGAMP did not impact on IFN production by H1975 cells (Fig. 7B), but increased type-I IFN production in macrophages (Fig. 7C), linking the enhanced antitumor phenotype with an increased type-I IFN macrophage response. These results suggest that STING agonists could hold some potential in favouring macrophage-mediated tumor elimination during osimertinib/anti-HER3 combination therapy, and further research in this area could address this question.

## Discussion

This study explores the urgent need for novel immunogenic treatments for advanced/metastatic mutant-EGFR/NSCLC patients, who currently receive the 3^rd^-generation TKI osimertinib as first-line treatment, and cannot receive ICI therapy for toxicity reasons, relying on standard chemotherapy beyond progression, without clear-cut stratification. We showed that osimertinib triggers apoptosis of NSCLC cells, but simultaneously upregulates another EGFR family member, HER3 through an IRE1α-requiring mechanism. In mice, osimertinib-treated tumors displayed high macrophage infiltration, therefore we combined osimertinib with anti-HER3 antibodies and studied the tumor/macrophage interplay. The combination treatment stimulated cGAS in cancer cells to produce cGAMP, which did not elicit type-I IFN production in tumor cells, but caused macrophages to produce type-I IFN. Macrophages contributed to eliminate double-treated tumor cells through FcγR-mediated effector activity, and this response was modulated by 2’3’-cGAMP. These results open new therapeutic avenues for NSCLC patients undergoing osimertinib treatment, not only through co-targeting HER3 but via engaging anti-tumor immunity, contributing to elimination of eventually resistant cells. Our study explores for the first time the innate immune component triggered by osimertinib in the context of targeting HER3 to treat TKI resistance in NSCLC, generating a rationale for trials aiming to improve anti-tumor immunity in mutant-EGFR/NSCLC patients.

Due to the intrinsic mutagenesis of NSCLC under treatment, TKI resistance is unavoidable. Historically, TKIs required reshaping to newer generations targeting novel mutations, illustrating the need for complementary therapies focused beyond the main mutation driver. Inhibition of EGFR family members can lead to compensatory activation of other tyrosine kinases [42], including ErbB family members such as HER3 [43, 44]. Reportedly, HER3 is upregulated by targeting HER2 [45, 46], and by targeting double-mutant EGFR^L858R–T790M^ with osimertinib [13, 14]. The rewiring mechanisms that cancers use to feed addictive oncogenic ErbB pathways have remained an investigation source without clear answers [47, 48], but appear to be both cancer-specific and treatment-specific [10, 49, 50]. This ambiguous nature of resistance links to the signalling variability of different types and stages of cancers, which adapt differently depending on treatment, triggering evolutive resistance mechanisms. Growing evidence points to tumor cells hijacking adaptive stress pathways [51]. These are responses that normal cells activate to either adapt to stress conditions or to initiate apoptosis if stress is beyond the salvage point, such as the ER stress-triggered UPR [7, 52, 53]. We observed IRE1α upregulation post-osimertinib, without involvement of the PERK or ATF6 branches of the UPR. Similarly cetuximab, targeting EGFR^WT^, has been linked to UPR activation in colorectal cancer cells [6]. Accordingly in that study, neither the PERK nor ATF6 branches were involved but interestingly, IRE1α inhibition was required for engaging immunogenic cell death in the BRAF-mutant setting, showing how IRE1α mediates tumor resistance through immune evasion. Whilst we did not see full IRE1α control over baseline HER3 levels, the response to osimertinib-induced stress was dependent on IRE1α, illustrating the complex evolutive mechanisms allowing tumor cells to rewire adaptive pathways in response to chemotherapy.

HER3 upregulation, whilst rendering cells more oncogenic, opens a window for therapeutic intervention using mAbs. This option has been explored pre-clinically and clinically. Concomitant to osimertinib, cell lines and mice were exposed to a combination of cetuximab (anti-EGFR), trastuzumab (anti-HER2) and NG33 (anti-HER3), showing responses in osimertinib-resistant models [13]. Mechanistically there, osimertinib triggered apoptosis similarly to our observations. However, the triple-mAb combination triggered cell cycle arrest preventing growth of resistant cells, whereas we observed that anti-HER3 mAbs MP-RM1 and EV20 engaged macrophage-dependent cytotoxicity. Using a different (ADC) approach, a pre-clinical study with HER3-DXd (patrutimab-deruxtecan) showed effectiveness in HER3-expressing patient-derived xenografts [54]. We observed that osimertinib-induced surface HER3-upregulation was required for antibody-mediated immune cytotoxicity against tumors. Similarly, pre-treating human xenografts or cells with osimertinib increased surface HER3-expression, augmenting susceptibility to HER3-DXd [54, 55]. These observations imply that timing between osimertinib-triggered HER3-upregulation and mAb treatment is an advantage. Accordingly, at the clinical level, HER3-DXd exhibits antitumor activity in patients with prior EGFR TKI therapy. In that study, 86% of patients had prior osimertinib therapy, and 91% of patients had prior platinum-based chemotherapy [14]. These studies and ours, confirm that HER3-targeting with mAbs offers a viable approach to decrease disease progression on TKI therapy. However, neither the triple-mAb combination or HER3-DXd have been shown to elicit STING-dependent immune responses.

It is accepted that long-lasting tumor control can only be achieved via engaging an immune response. Adaptive responses require innate immunity for activation, maturation, and memory [56]. Amongst the studies targeting HER3 in TKI-resistant NSCLC, ours is the first focused on the innate immune component triggered by osimertinib. We observed high macrophage infiltration towards HER3-high areas post-osimertinib. Thus, we targeted the extracellular domain of HER3, activating FcγR-dependent macrophage-mediated tumor cytotoxicity. For mice or murine-BMDMs we used MP-RM1 (murine IgG2a), whereas for human PBMCs or humanised-FcγR BMDMs from chimeric mice [38], we used EV20 (human IgG1) based on Fc-domain/FcγR compatibility. The cytotoxic response was higher using EV20 than MP-RM1, which we attribute to the more abundant landscape of activatory FcγRs (ITAM-coupled) in humans compared to mice [57]. This suggests that in patients we might expect to see a profound anti-tumor response using our combination strategy. These results importantly offer an immune-engaging alternative compared to HER3-ADCs, as FcγR-mediated responses can impact beyond direct tumor elimination by effector cells, through eliciting adaptive long-lasting anti-tumor vaccination effects [58].

An important barrier when activating tumoral macrophage responses is overcoming their immunosuppressive phenotype to elicit antitumor activity [59, 60]. In this regard, STING and type-I IFN responses are crucial stimulating an inflammatory macrophage phenotype [56]. The DNA sensor cGAS can be activated in a tumor-autonomous way by chromosomal instability leading to cancer-cell STING activation [61], but can also lead to cGAMP transfer to surrounding immune cells, activating STING in trans, leading to type-I IFN production in antigen presenting cells and importantly, in macrophages [40, 41]. Combining xenograft immunofluorescence studies and state-of-the-art FRET FLIM techniques, we observed that osimertinib treatment, non-autonomously activated cGAS leading to cGAMP production in tumor cells, activating STING in TAMs. Although we did not measure extracellular cGAMP, our human tumor cell/M1-like macrophage co-culture model allowed us to clearly differentiate between cancer cell-derived *vs*. macrophage-derived type-I IFN production in response to tumoral cGAMP production. This allowed us to validate other observations describing tumoral cGAS transactivating STING in immune cells [40, 41, 62, 63]. In agreement with this, the external addition of 2’3’-cGAMP enhanced the macrophage-dependent antitumor response in our in vitro setting. Future studies using targeted STING agonists would assess the benefits of enhancing an anti-tumor innate response to this combination therapy, an interesting opportunity considering the number of STING agonists being tested currently in clinical trials.

Evidence indicates that female NSCLC patients display higher exon-21 L858R mutation frequency than males [64, 65], and increased frequency of lung adenocarcinomas depending on ethnicity [66]. The EGFR^L858R–T790M^ double-mutant H1975 line originates from a female patient’s primary NSCLC, hence we used female mice for in vivo experiments. Despite this experimental limitation, we have found no evidence of sex-dependent differences between immune responses in subcutaneous cancer models with CD1-nude mice. However, female mice do offer behavioural advantages that could otherwise translate into experimental artifacts, as male mice housed together naturally show dominance which can cause injuries between littermates producing wound-induced inflammation artifacts, affecting studies on immune infiltration.

In summary, osimertinib combined with anti-HER3 antibodies elicits an innate immune response modulated by STING agonist 2’3-cGAMP. Triple-antibody therapy targeting HER1-2-3, although previously demonstrated strong tumor control, raises the risk of potential long-term adverse effects such as chronic heart failure [67, 68], given that EGFR, HER2 and HER3 are expressed in myocardium [69, 70]. Likewise, HER2-ADCs do exhibit cardiotoxicity [71], illustrating that myocardium is a target for payload delivery. The opportunity to target the triplet of tumor-restricted mutant-EGFR^L858R–T790M^, over-expressed HER3 and TAM-STING, offers the wider advantage to be more tumor-specific and immunogenic, and therefore less harmful long-term to non-tumoral tissues. We additionally suggest that patients could be stratified based on HER3 levels during response to osimertinib treatment. Stratification may facilitate prospective testing of patient cohorts to more accurately select individuals that would best respond to an osimertinib/anti-HER3 therapeutic combination.

## Supplementary information

Reproducibility checklist

Suppl Fig Legends

Supplementary Figure S1

Supplementary Figure S2

Supplementary Figure S3

Supplementary Figure S4

Supplementary Figure S5

Supplementary Figure S6

Supplementary Figure S7

## Data Availability

All data needed to evaluate the conclusions in the paper are present in the paper. Additional data related to this paper may be requested from the corresponding author.
