# Supplementary material for: Osimertinib and anti-HER3 combination therapy engages immune dependent tumor toxicity via STING activation in trans
Source: Cell Death Dis. 2022 Mar 28;13(3):274. doi: 10.1038/s41419-022-04701-3 (PMC8960767; doi:10.1038/s41419-022-04701-3)
Supplement: Supplementary file 2 — Suppl Fig Legends [file 41419_2022_4701_MOESM2_ESM.docx]

**Supplementary Figure Legends**

**Supplementary Figure S1**

**A-D.** H1975 cells (EGFR^L585R–T790M^) and A549 cells (EGFR^WT^) were treated with osimertinib (Osi) or cisplatin (Cis) at the indicated concentrations for 24 h; apoptosis was measured using flow cytometry with DiOC_6_(3) for mitochondrial transmembrane potential (ΔΨ_m_) and PI for viability, as described in materials and methods. Representative dot plots show vehicle *vs.* treated cells as indicated; the graphs show means ± SEM of three replicate experiments. ***P*<0.01 *vs.* vehicle (0 μM) as indicated, using unpaired *t*-test with total apoptotic values (combined ΔΨ_m_^–^ PI^–^ and ΔΨ_m_^–^ PI^+^ populations).

**E.** Western blot analysis from H1975 cells treated for 24 h with 200 nM osimertinib. Relative levels of HER3 relative to GAPDH were quantified from 3 independent replicates, shown under the representative blots as normalized mean values (a.u.); ***P*<0.01 *vs.* vehicle as revealed by unpaired *t*-test from 3 independent replicates.

**Supplementary Figure S2**

**A.** Cell sorting gating strategy for obtaining Cas9/CRISPR stable non-targeting control (NTC) and IRE1α-ko H1975 cells. H1975 cells were electroporated with Cas9 protein, tracRNA, crRNAs against ERN1 or NTC, and mCherry plasmid as detailed in materials and methods. After 72 h, cells were resuspended and sorted using a BD Aria Fusion. Parental, non-electroporated H1975 cells were used as a negative gate control (left panel). Fluorescence of mCherry was used to gate on positively electroporated cells, selecting a live population (Dapi^Neg^) with mild mCherry expression, excluding auto-fluorescent cells indicated for each condition (lower panels); 96 well plates were obtained with single clones for further growth, expansion, and selection.

**B.** Cas9/CRISPR stable non-targeting control (NTC) and IRE1α-ko H1975 clonal cells were assessed for loss of IRE1α after clonal expansion, using near-IR western blot. crNTC clones #1 & #2 and crIRE1α clones #4 & #5 were chosen for further crIRE1α-ko functional assessment.

**C.** Extended view of Figure 2B. Parental H1975 cells, or Cas9/CRISPR stable non-targeting control (NTC) and IRE1α-ko H1975 cells were treated with ER-stressor tunicamycin (1 μg/ml) for 3 h to assess functionality of IRE1α. Parental and NTC H1975 cells responded to tunicamycin displaying IRE1α functionality (producing Xbp1s); both IRE1α-ko clone candidates displayed lack of IRE1α functionality. Clone #4 was used in further experiments.

**Supplementary Figure S3**

**A.** H1975 cells pre-treated with osimertinib (Osi, 200 nM, 24 h) were treated with anti-HER3 (MP-RM-1, 10 μg/ml, 1 h) and then co-cultured with M0, M1-like or M2-like BMDMs coming from CD1 nude mice (2 h, ratio 10:1) pre-labelled with cell tracker. Apoptosis of H1975 cells exclusively was assessed by flow cytometry, excluding BMDMs as described in materials and methods. Dot plots are representative of 3 independent experiments.

**B.** Extended view from Fig Quantification summary from the representative data shown in panel A. H1975 cells alone or co-cultured with BMDM from CD1 nude mice were assessed for apoptosis using flow cytometry with DiOC_6_(3) for mitochondrial transmembrane potential (ΔΨ_m_) and Dapi for cell viability, as described in materials and methods. Results shown as mean ± SEM from three replicates; ***P*<0.01 as indicated, using one-way ANOVA with Tukey’s test, calculated using total apoptotic values

**Supplementary Figure S4**

**A.** H1975 cells pre-treated with osimertinib (Osi, 200 nM, 24 h) were treated with human-Fc anti-HER3 (^Hu^Anti-HER3, EV20; 10 μg/ml, 1 h) and then then co-cultured with M0, M1-like or M2-like BMDMs coming from chimeric humanised Fc receptor (^Hu^FcγR) mice (2 h, ratio 10:1 BMDM:H1975) pre-labelled with cell tracker. Apoptosis of H1975 cells was assessed by flow cytometry excluding macrophages as described in materials and methods. Dot plots are representative of 3 independent replicates.

**B.** Quantification summary (extended view from Fig. 3D) from the representative data shown in panel A. H1975 cells alone or co-cultured with M0, M1-like or M2-like BMDMs from humanised Fc receptor (Hu-FcγR) mice were assessed for apoptosis using flow cytometry with DiOC_6_(3) for mitochondrial transmembrane potential (ΔΨ_m_) and Dapi for cell viability, as described in materials and methods. Results shown as mean ± SEM from three replicates; ***P*<0.01 as indicated, using one-way ANOVA with Tukey’s test.

**C.** Extended view data from Fig. 3E. Cas9/CRISPR stable NTC or IRE1α-ko H1975 cells pre-treated with osimertinib (200 nM, 24 h) were treated with murine anti-HER3 (MP-RM-1, 10 μg/ml, 1 h) and then co-cultured with BMDM from CD1 nude mice (M1; 2 h, ratio 10:1) pre-labelled with cell tracker. Apoptosis of H1975 cells was assessed by flow cytometry excluding macrophages as described in materials and methods. Plots are representative of 3 independent replicates (left panel). Data are shown as mean ± SEM. ***P*<0.01 as indicated, using unpaired *t*-test with total values (right panel).

**Supplementary Figure S5**

**A.** Quantification of CD11b^+^ immune infiltration using flow cytometry from tumors dissected from treatment groups defined in Fig. 4A. Data shown as floating boxes (mean ± interval) including datapoints. One-way ANOVA with Tukey’s multiple group comparison test revealed no significant differences between Osi and Osi + Anti-HER3.

**B.** Confocal images from *in vivo* tumor slices, showing cGAS puncta (green), STING (red) and DAPI (blue). Representative images from 3 tumor stains; white scalebars as indicated in the magnified images corresponding to the whit boxes indicated in the left images. The right side shows line-scan analysis with fluorescence intensities across the 250-μm-long white arrows from the cGAS channel. The inner *vs.* outer zones of tumors are shown as pale *vs.* darker gray respectively, showing increased cGAS foci in the inner zone of tumors after osimertinib treatment. Graphs are representative from 3 replicate tumors.

**Supplementary Figure S6**

**A.** Extended view of Figure 5A. Confocal immunofluorescence images from xenograft tumor slices, showing cGAS (red) distribution in diffuse puncta in Vehicle or Anti-HER3 conditions, which aggregates to form foci in Osi or Osi + Anti-HER3 conditions; infiltrating macrophages (F4/80, green) which increase in Osi and Osi + Anti-HER3 conditions, STING (white) is not detectable in macrophages under Vehicle conditions, increasing in Osi and Osi + Anti-HER3 conditions and is not present in macrophages in Anti-HER3 conditions, but in other cells without a clear perinuclear pattern as seen for Osi or Osi + Anti-HER3; nuclei (Dapi, blue). Images are representative from treatment groups indicated (defined in Figure 4A). White scalebars are indicated in merged channel images.

**B.** Data from Fig. 5B, shown here for clarity comparing all conditions from panel A. Infiltrating macrophages (F4/80^+^) were quantified *vs.* total cell number (Dapi) using Fiji-ImageJ. Data shown as mean ± SEM from image fields corresponding to the treatment groups indicated (defined in Figure 4A). Statistical analysis using one-way ANOVA with Tukey’s multiple group comparison test revealed **P*<0.05 and ****P*<0.001 relative to vehicle or as indicated.

**C.** Data from Fig. 5C, shown here for clarity comparing all conditions from panel A. STING-positive macrophages (STING^+^ and F4/80^+^) were quantified *vs.* total macrophage number (F4/80^+^) using Fiji-ImageJ. Data shown as mean ± SEM from 4 independent image fields coming from the treatment groups indicated (defined in Figure 4A). Statistical analysis using one-way ANOVA with Tukey’s multiple group comparison test revealed ****P*<0.001 relative to vehicle or **P*<0.05 and as indicated.

**Supplementary Figure S7**

**A.** Full-size images from the western blots show in Fig 2A.

**B.** Full-size images from the western blots show in Fig 2B.

**C.** Full-size images from the western blots show in Fig 2C.
