# Supplementary figures and images for: Osimertinib and anti-HER3 combination therapy engages immune dependent tumor toxicity via STING activation in trans
Source: Cell Death Dis. 2022 Mar 28;13(3):274. doi: 10.1038/s41419-022-04701-3 (PMC8960767; doi:10.1038/s41419-022-04701-3)

Supplementary figure S1

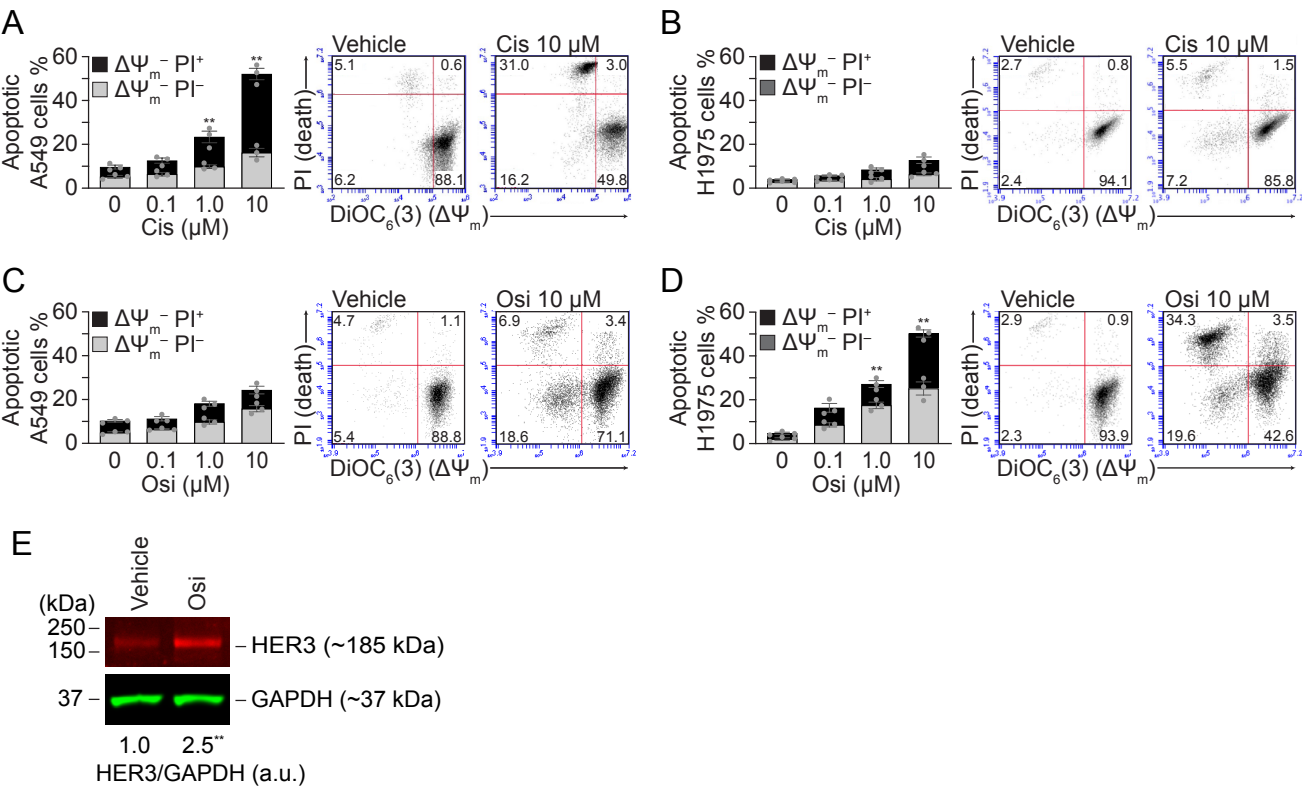

Supplement: Supplementary file 3 — Supplementary Figure S1 [file 41419_2022_4701_MOESM3_ESM.pdf]

## A

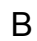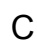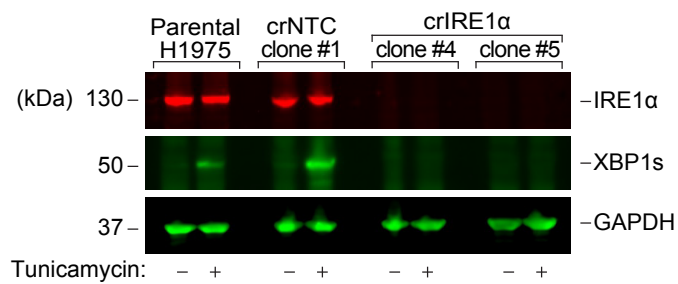

Supplement: Supplementary file 4 — Supplementary Figure S2 [file 41419_2022_4701_MOESM4_ESM.pdf]

Supplmentary figure S3

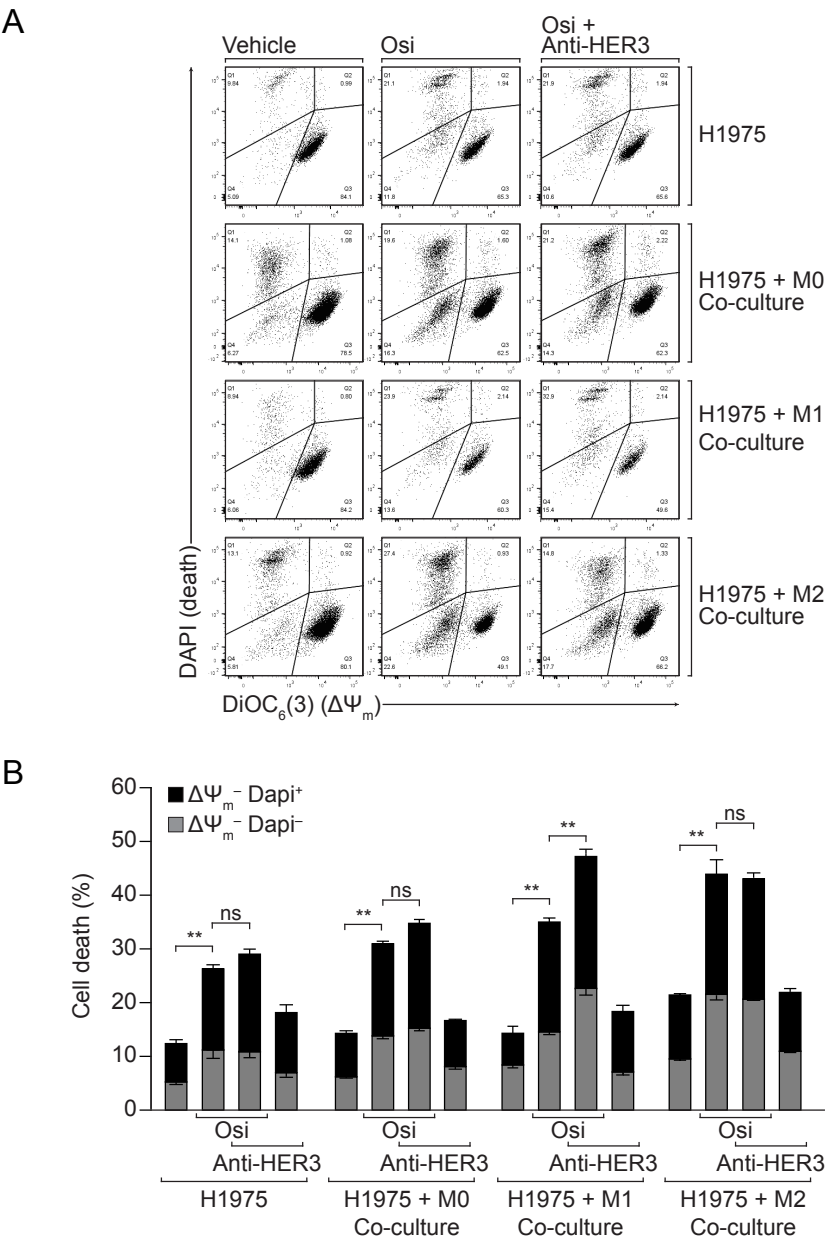

Supplement: Supplementary file 5 — Supplementary Figure S3 [file 41419_2022_4701_MOESM5_ESM.pdf]

Supplimentary figure S4

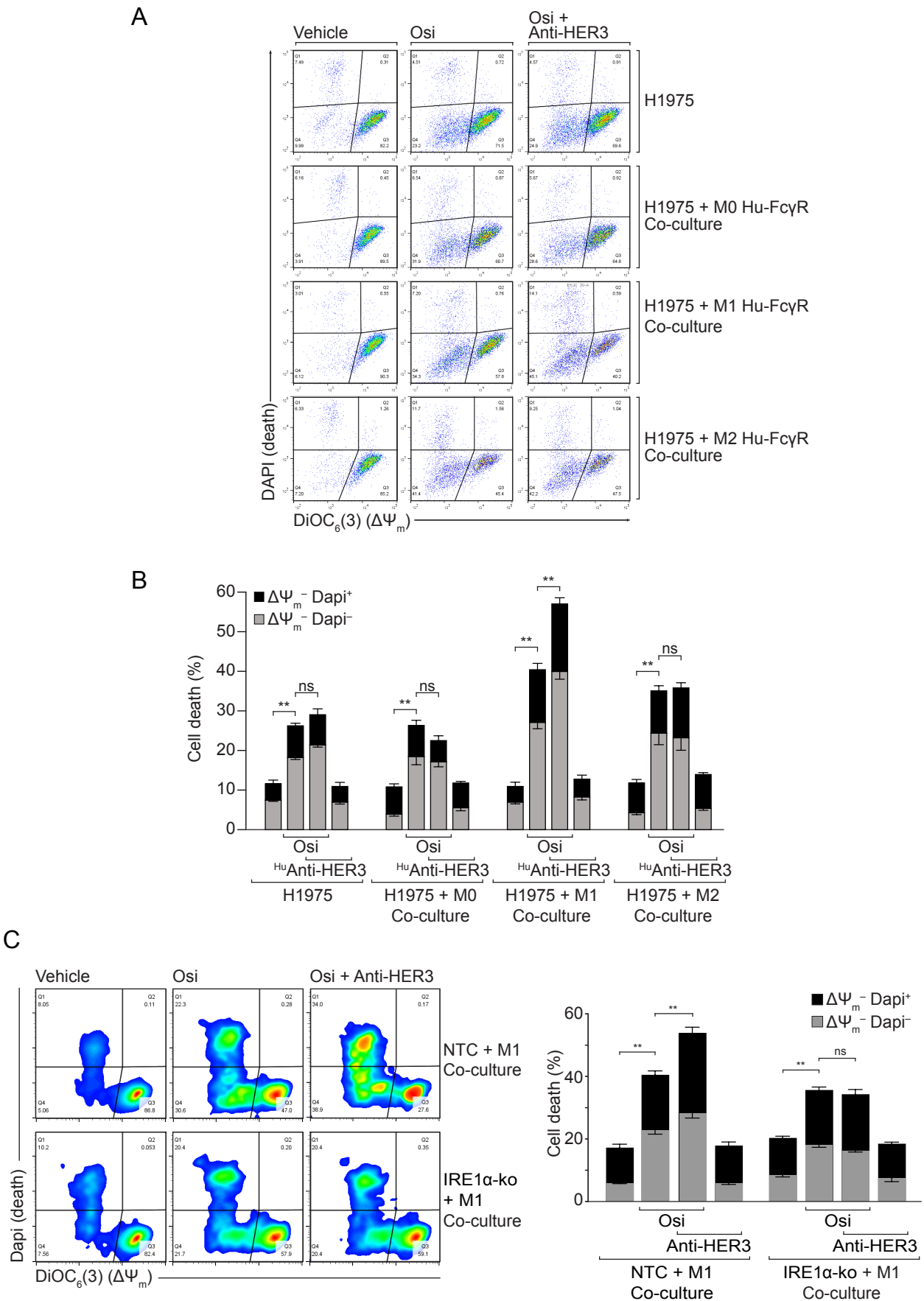

Supplement: Supplementary file 6 — Supplementary Figure S4 [file 41419_2022_4701_MOESM6_ESM.pdf]

Supplementary figure S5

A

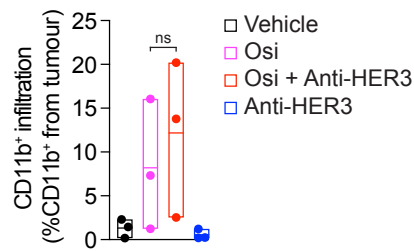

B

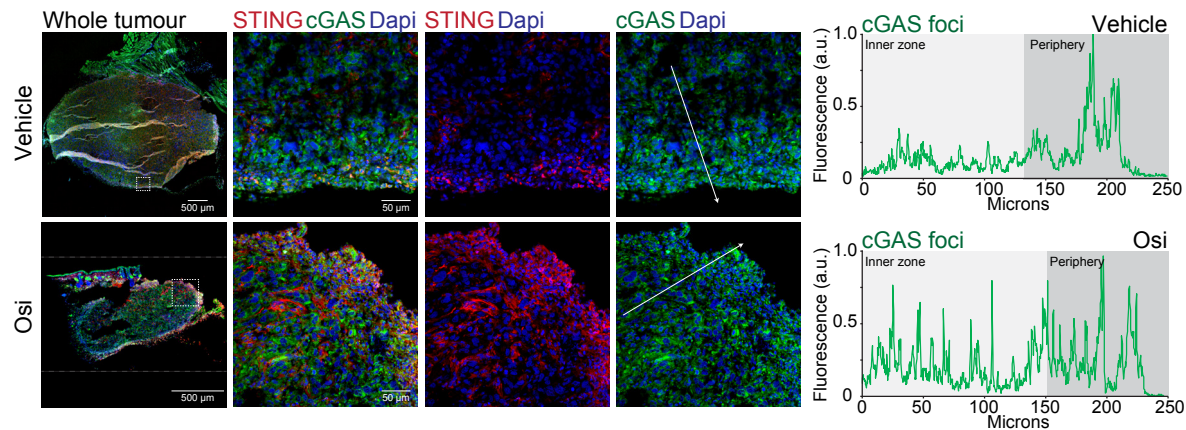

Supplement: Supplementary file 7 — Supplementary Figure S5 [file 41419_2022_4701_MOESM7_ESM.pdf]

Supplementary figure S6

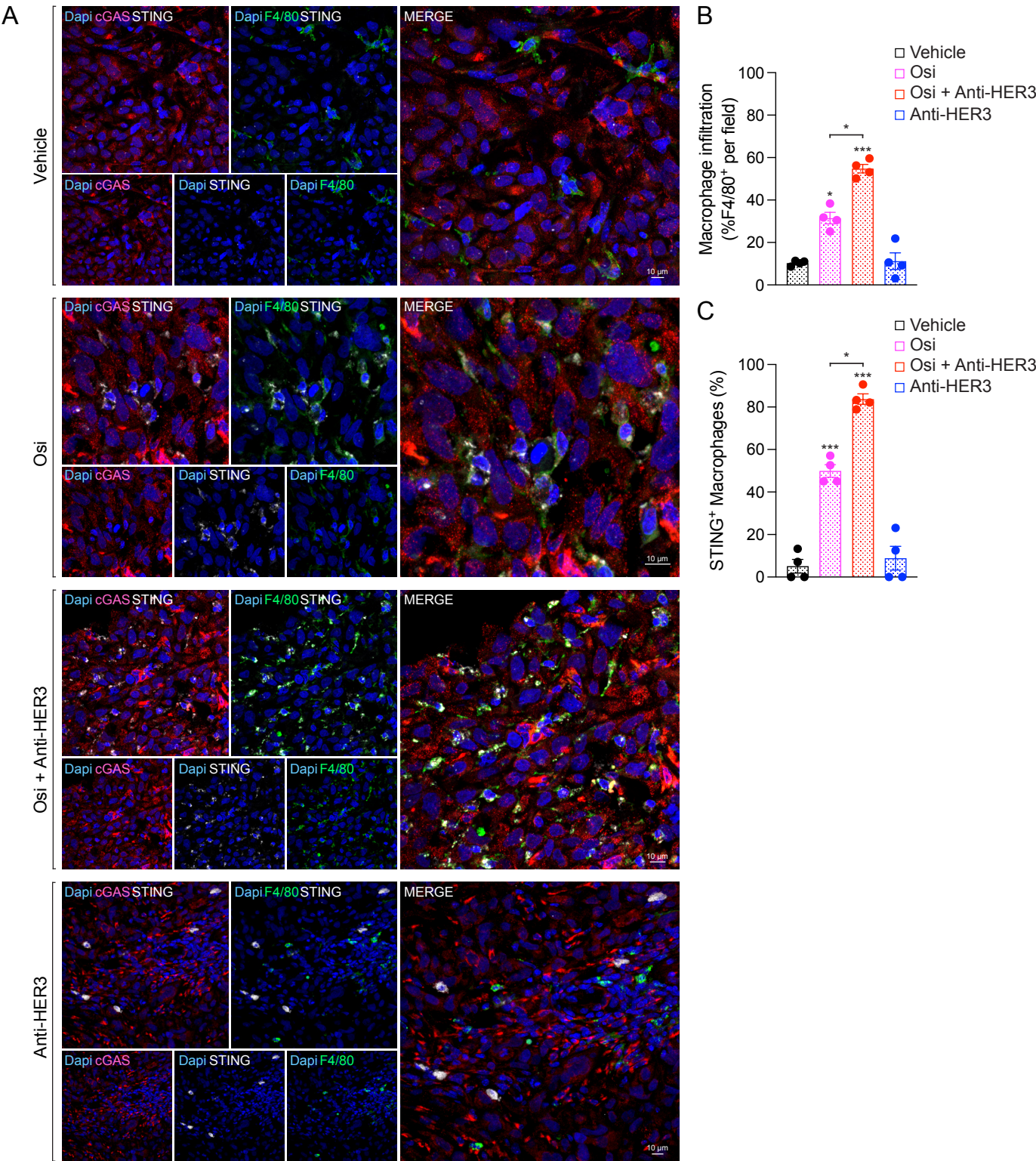

Supplement: Supplementary file 8 — Supplementary Figure S6 [file 41419_2022_4701_MOESM8_ESM.pdf]
