# Supplementary material for: Osimertinib and anti-HER3 combination therapy engages immune dependent tumor toxicity via STING activation in trans
Source: Cell Death Dis. 2022 Mar 28;13(3):274. doi: 10.1038/s41419-022-04701-3 (PMC8960767; doi:10.1038/s41419-022-04701-3)

Supplmentary figure S7

A Full-size blots from Figure 2A

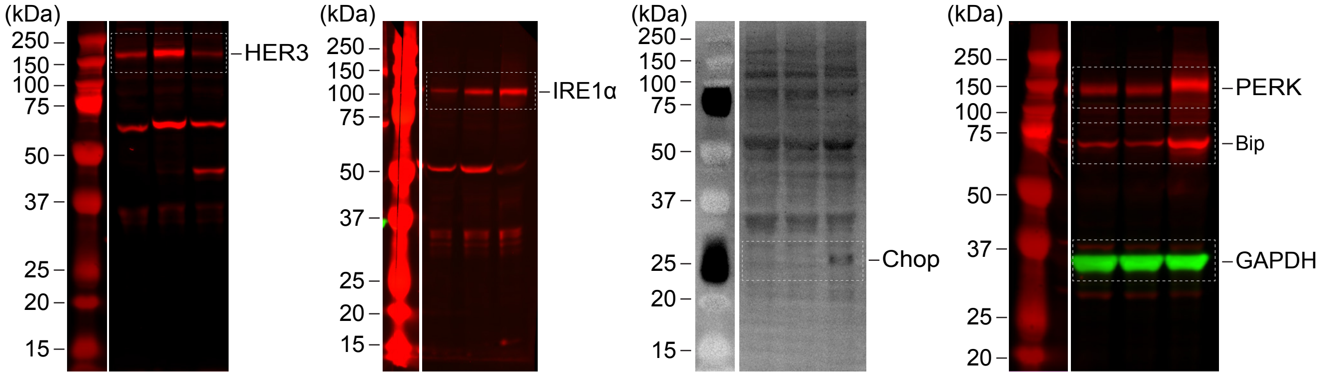

B Full-size blots from Figure 2B

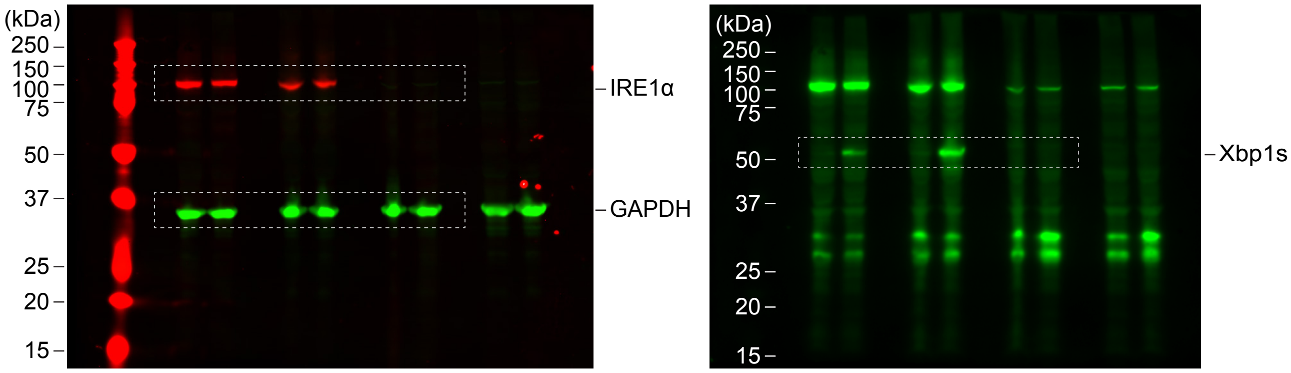

C Full-size blots from Figure 2C

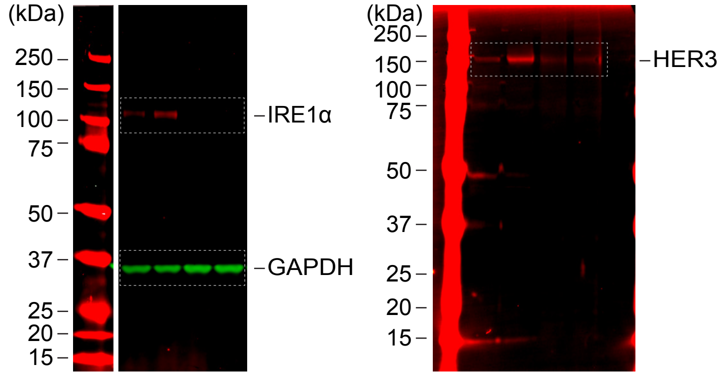

Supplement: Supplementary file 9 — Supplementary Figure S7 [file 41419_2022_4701_MOESM9_ESM.pdf]
